# Supplementary material for: Discovery and application of insertion-deletion (INDEL) polymorphisms for QTL mapping of early life-history traits in Atlantic salmon
Source: BMC Genomics. 2010 Mar 8;11:156. doi: 10.1186/1471-2164-11-156 (PMC2838853; doi:10.1186/1471-2164-11-156)
Supplement: Additional file 2 — Information on developed 76 locus single-run INDEL panel in Atlantic salmon. Information on fluorescence labeling, primer concentrations, PCR pooling and links to alignments, INDEL motifs and GENESCAN (Burge and Karlin 1997) predictions of genes/exons are available in html format. [file 1471-2164-11-156-S2.ZIP › Additionalfile2/table4707.html]

Contig Alignment

```
Base	A B C D E F G H I J K L M N O P Q R S T U V W X Y Z a b c d e f g   
1	G . . . . . . . . . . . . . . . . . . . . . . . . . . . . . . . .    
2	G . . . . . . . . . . . . . . . . . . . . . . . . . . . . . . . .    
3	G . . . . . . . . . . . . . . . . . . . . . . . . . . . . . . . .    
4	G . . . . . . . . . . . . . . . . . . . . . . . . . . . . . . . .    
5	A . . . . . . . . . . . . . . . . . . . . . . . . . . . . . . . .    
6	G . . . . . . . . . . . . . . . . . . . . . . . . . . . . . . . .    
7	A . . . . . . . . . . . . . . . . . . . . . . . . . . . . . . . .    
8	G . . . . . . . . . . . . . . . . . . . . . . . . . . . . . . . .    
9	G . . . . . . . . . . . . . . . . . . . . . . . . . . . . . . . .    
10	A . . . . . . . . . . . . . . . . . . . . . . . . . . . . . . . .    
11	A . . . . . . . . . . . . . . . . . . . . . . . . . . . . . . . .    
12	T . . . . . . . . . . . . . . . . . . . . . . . . . . . . . . . .    
13	C . . . . . . . . . . . . . . . . . . . . . . . . . . . . . . . .    
14	G . . . . . . . . . . . . . . . . . . . . . . . . . . . . . . . .    
15	T . . . . . . . . . . . . . . . . . . . . . . . . . . . . . . . .    
16	A . . . . . . . . . . . . . . . . . . . . . . . . . . . . . . . .    
17	A . . . . . . . . . . . . . . . . . . . . . . . . . . . . . . . .    
18	G . . . . . . . . . . . . . . . . . . . . . . . . . . . . . . . .    
19	T . . . . . . . . . . . . . . . . . . . . . . . . . . . . . . . .    
20	T . . . . . . . . . . . . . . . . . . . . . . . . . . . . . . . .    
21	T . . . . . . . . . . . . . . . . . . . . . . . . . . . . . . . .    
22	T . . . . . . . . . . . . . . . . . . . . . . . . . . . . . . . .    
23	C . . . . . . . . . . . . . . . . . . . . . . . . . . . . . . . .    
24	C . . . . . . . . . . . . . . . . . . . . . . . . . . . . . . . .    
25	A . . . . . . . . . . . . . . . . . . . . . . . . . . . . . . . .    
26	T . . . . . . . . . . . . . . . . . . . . . . . . . . . . . . . .    
27	A . . . . . . . . . . . . . . . . . . . . . . . . . . . . . . . .    
28	G . . . . . . . . . . . . . . . . . . . . . . . . . . . . . . . .    
29	T . . . . . . . . . . . . . . . . . . . . . . . . . . . . . . . .    
30	G . . . . . . . . . . . . . . . . . . . . . . . . . . . . . . . .    
31	A . . . . . . . . . . . . . . . . . . . . . . . . . . . . . . . .    
32	T . . . . . . . . . . . . . . . . . . . . . . . . . . . . . . . .    
33	C . . . . . . . . . . . . . . . . . . . . . . . . . . . . . . . .    
34	T . . . . . . . . . . . . . . . . . . . . . . . . . . . . . . . .    
35	A . . . . . . . . . . . . . . . . . . . . . . . . . . . . . . . .    
36	G . . . . . . . . . . . . . . . . . . . . . . . . . . . . . . . .    
37	A . . . . . . . . . . . . . . . . . . . . . . . . . . . . . . . .    
38	G . . . . . . . . . . . . . . . . . . . . . . . . . . . . . . . .    
39	G . . . . . . . . . . . . . . . . . . . . . . . . . . . . . . . .    
40	G . . . . . . . . . . . . . . . . . . . . . . . . . . . . . . . .    
41	C . . . . . . . . . . . . . . . . . . . . . . . . . . . . . . . .    
42	A . . . . . . . . . . . . . . . . . . . . . . . . . . . . . . . .    
43	G . . . . . . . . . . . . . . . . . . . . . . . . . . . . . . . .    
44	G . . . . . . . . . . . . . . . . . . . . . . . . . . . . . . . .    
45	G . . . . . . . . . . . . . . . . . . . . . . . . . . . . . . . .    
46	G . . . . . . . . . . . . . . . . . . . . . . . . . . . . . . . .    
47	A . . . . . . . . . . . . . . . . . . . . . . . . . . . . . . . .    
48	T . . . . . . . . . . . . . . . . . . . . . . . . . . . . . . . .    
49	G . . . . . . . . . . . . . . . . . . . . . . . . . . . . . . . .    
50	A . . . . . . . . . . . . . . . . . . . . . . . . . . . . . . . .    
51	T . . . . . . . . . . . . . . . . . . . . . . . . . . . . . . . .    
52	G G . . . . . . . . . . . . . . . . . . . . . . . . . . . . . . .    
53	G G . . . . . . . . . . . . . . . . . . . . . . . . . . . . . . .    
54	A A . . . . . . . . . . . . . . . . . . . . . . . . . . . . . . .    
55	C C . . . . . . . . . . . . . . . . . . . . . . . . . . . . . . .    
56	C C . . . . . . . . . . . . . . . . . . . . . . . . . . . . . . .    
57	C C . . . . . . . . . . . . . . . . . . . . . . . . . . . . . . .    
58	A A . . . . . . . . . . . . . . . . . . . . . . . . . . . . . . .    
59	A A . . . . . . . . . . . . . . . . . . . . . . . . . . . . . . .    
60	T T . . . . . . . . . . . . . . . . . . . . . . . . . . . . . . .    
61	A A . . . . . . . . . . . . . . . . . . . . . . . . . . . . . . .    
62	T T . . . . . . . . . . . . . . . . . . . . . . . . . . . . . . .    
63	T T . . . . . . . . . . . . . . . . . . . . . . . . . . . . . . .    
64	T T . . . . . . . . . . . . . . . . . . . . . . . . . . . . . . .    
65	T T . . . . . . . . . . . . . . . . . . . . . . . . . . . . . . .    
66	G G . . . . . . . . . . . . . . . . . . . . . . . . . . . . . . .    
67	A A . . . . . . . . . . . . . . . . . . . . . . . . . . . . . . .    
68	A A . . . . . . . . . . . . . . . . . . . . . . . . . . . . . . .    
69	C C . . . . . . . . . . . . . . . . . . . . . . . . . . . . . . .    
70	G G . . . . . . . . . . . . . . . . . . . . . . . . . . . . . . .    
71	A A . . . . . . . . . . . . . . . . . . . . . . . . . . . . . . .    
72	T T . . . . . . . . . . . . . . . . . . . . . . . . . . . . . . .    
73	G G . . . . . . . . . . . . . . . . . . . . . . . . . . . . . . .    
74	A A . . . . . . . . . . . . . . . . . . . . . . . . . . . . . . .    
75	A A . . . . . . . . . . . . . . . . . . . . . . . . . . . . . . .    
76	G G . . . . . . . . . . . . . . . . . . . . . . . . . . . . . . .    
77	C C . . . . . . . . . . . . . . . . . . . . . . . . . . . . . . .    
78	C C . . . . . . . . . . . . . . . . . . . . . . . . . . . . . . .    
79	A A . . . . . . . . . . . . . . . . . . . . . . . . . . . . . . .    
80	T T . . . . . . . . . . . . . . . . . . . . . . . . . . . . . . .    
81	T T . . . . . . . . . . . . . . . . . . . . . . . . . . . . . . .    
82	T T . . . . . . . . . . . . . . . . . . . . . . . . . . . . . . .    
83	T T . . . . . . . . . . . . . . . . . . . . . . . . . . . . . . .    
84	T T . . . . . . . . . . . . . . . . . . . . . . . . . . . . . . .    
85	T T . . . . . . . . . . . . . . . . . . . . . . . . . . . . . . .    
86	G G . . . . . . . . . . . . . . . . . . . . . . . . . . . . . . .    
87	T T . . . . . . . . . . . . . . . . . . . . . . . . . . . . . . .    
88	A A . . . . . . . . . . . . . . . . . . . . . . . . . . . . . . .    
89	A A . . . . . . . . . . . . . . . . . . . . . . . . . . . . . . .    
90	A A . . . . . . . . . . . . . . . . . . . . . . . . . . . . . . .    
91	A A . . . . . . . . . . . . . . . . . . . . . . . . . . . . . . .    
92	A A . . . . . . . . . . . . . . . . . . . . . . . . . . . . . . .    
93	A A . . . . . . . . . . . . . . . . . . . . . . . . . . . . . . .    
94	A A . . . . . . . . . . . . . . . . . . . . . . . . . . . . . . .    
95	T T . . . . . . . . . . . . . . . . . . . . . . . . . . . . . . .    
96	T T . . . . . . . . . . . . . . . . . . . . . . . . . . . . . . .    
97	T T . . . . . . . . . . . . . . . . . . . . . . . . . . . . . . .    
98	C C . . . . . . . . . . . . . . . . . . . . . . . . . . . . . . .    
99	C C . . . . . . . . . . . . . . . . . . . . . . . . . . . . . . .    
100	C C . . . . . . . . . . . . . . . . . . . . . . . . . . . . . . .    
101	C C . . . . . . . . . . . . . . . . . . . . . . . . . . . . . . .    
102	C C . . . . . . . . . . . . . . . . . . . . . . . . . . . . . . .    
103	A A . . . . . . . . . . . . . . . . . . . . . . . . . . . . . . .    
104	A A . . . . . . . . . . . . . . . . . . . . . . . . . . . . . . .    
105	C C . . . . . . . . . . . . . . . . . . . . . . . . . . . . . . .    
106	T T . . . . . . . . . . . . . . . . . . . . . . . . . . . . . . .    
107	A A . . . . . . . . . . . . . . . . . . . . . . . . . . . . . . .    
108	A A . . . . . . . . . . . . . . . . . . . . . . . . . . . . . . .    
109	A A . . . . . . . . . . . . . . . . . . . . . . . . . . . . . . .    
110	T T . . . . . . . . . . . . . . . . . . . . . . . . . . . . . . .    
111	T T . . . . . . . . . . . . . . . . . . . . . . . . . . . . . . .    
112	T T . . . . . . . . . . . . . . . . . . . . . . . . . . . . . . .    
113	G G . . . . . . . . . . . . . . . . . . . . . . . . . . . . . . .    
114	C C . . . . . . . . . . . . . . . . . . . . . . . . . . . . . . .    
115	T T . . . . . . . . . . . . . . . . . . . . . . . . . . . . . . .    
116	C C . . . . . . . . . . . . . . . . . . . . . . . . . . . . . . .    
117	A A . . . . . . . . . . . . . . . . . . . . . . . . . . . . . . .    
118	A A . . . . . . . . . . . . . . . . . . . . . . . . . . . . . . .    
119	T T . . . . . . . . . . . . . . . . . . . . . . . . . . . . . . .    
120	A A . . . . . . . . . . . . . . . . . . . . . . . . . . . . . . .    
121	A A . . . . . . . . . . . . . . . . . . . . . . . . . . . . . . .    
122	T T . . . . . . . . . . . . . . . . . . . . . . . . . . . . . . .    
123	G G . . . . . . . . . . . . . . . . . . . . . . . . . . . . . . .    
124	G G . . . . . . . . . . . . . . . . . . . . . . . . . . . . . . .    
125	C C . . . . . . . . . . . . . . . . . . . . . . . . . . . . . . .    
126	C C . . . . . . . . . . . . . . . . . . . . . . . . . . . . . . .    
127	A A . . . . . . . . . . . . . . . . . . . . . . . . . . . . . . .    
128	T T . . . . . . . . . . . . . . . . . . . . . . . . . . . . . . .    
129	T T . . . . . . . . . . . . . . . . . . . . . . . . . . . . . . .    
130	T T . . . . . . . . . . . . . . . . . . . . . . . . . . . . . . .    
131	G G . . . . . . . . . . . . . . . . . . . . . . . . . . . . . . .    
132	C C . . . . . . . . . . . . . . . . . . . . . . . . . . . . . . .    
133	T T . . . . . . . . . . . . . . . . . . . . . . . . . . . . . . .    
134	G G . . . . . . . . . . . . . . . . . . . . . . . . . . . . . . .    
135	A A . . . . . . . . . . . . . . . . . . . . . . . . . . . . . . .    
136	A A . . . . . . . . . . . . . . . . . . . . . . . . . . . . . . .    
137	T T . . . . . . . . . . . . . . . . . . . . . . . . . . . . . . .    
138	T T . . . . . . . . . . . . . . . . . . . . . . . . . . . . . . .    
139	C C . . . . . . . . . . . . . . . . . . . . . . . . . . . . . . .    
140	C C . . . . . . . . . . . . . . . . . . . . . . . . . . . . . . .    
141	A A . . . . . . . . . . . . . . . . . . . . . . . . . . . . . . .    
142	A A . . . . . . . . . . . . . . . . . . . . . . . . . . . . . . .    
143	A A . . . . . . . . . . . . . . . . . . . . . . . . . . . . . . .    
144	T T . . . . . . . . . . . . . . . . . . . . . . . . . . . . . . .    
145	A A . . . . . . . . . . . . . . . . . . . . . . . . . . . . . . .    
146	A A . . . . . . . . . . . . . . . . . . . . . . . . . . . . . . .    
147	T T . . . . . . . . . . . . . . . . . . . . . . . . . . . . . . .    
148	G G . . . . . . . . . . . . . . . . . . . . . . . . . . . . . . .    
149	T T . . . . . . . . . . . . . . . . . . . . . . . . . . . . . . .    
150	A A . . . . . . . . . . . . . . . . . . . . . . . . . . . . . . .    
151	A A . . . . . . . . . . . . . . . . . . . . . . . . . . . . . . .    
152	T T . . . . . . . . . . . . . . . . . . . . . . . . . . . . . . .    
153	A A . . . . . . . . . . . . . . . . . . . . . . . . . . . . . . .    
154	T T . . . . . . . . . . . . . . . . . . . . . . . . . . . . . . .    
155	C C . . . . . . . . . . . . . . . . . . . . . . . . . . . . . . .    
156	T T . . . . . . . . . . . . . . . . . . . . . . . . . . . . . . .    
157	A A . . . . . . . . . . . . . . . . . . . . . . . . . . . . . . .    
158	C C . . . . . . . . . . . . . . . . . . . . . . . . . . . . . . .    
159	T T . . . . . . . . . . . . . . . . . . . . . . . . . . . . . . .    
160	G G . . . . . . . . . . . . . . . . . . . . . . . . . . . . . . .    
161	G G . . . . . . . . . . . . . . . . . . . . . . . . . . . . . . .    
162	G G . . . . . . . . . . . . . . . . . . . . . . . . . . . . . . .    
163	G G . . . . . . . . . . . . . . . . . . . . . . . . . . . . . . .    
164	T T . . . . . . . . . . . . . . . . . . . . . . . . . . . . . . .    
165	G G . . . . . . . . . . . . . . . . . . . . . . . . . . . . . . .    
166	T T . . . . . . . . . . . . . . . . . . . . . . . . . . . . . . .    
167	C C . . . . . . . . . . . . . . . . . . . . . . . . . . . . . . .    
168	T T . . . . . . . . . . . . . . . . . . . . . . . . . . . . . . .    
169	G G . . . . . . . . . . . . . . . . . . . . . . . . . . . . . . .    
170	T T . . . . . . . . . . . . . . . . . . . . . . . . . . . . . . .    
171	G G . . . . . . . . . . . . . . . . . . . . . . . . . . . . . . .    
172	G G . . . . . . . . . . . . . . . . . . . . . . . . . . . . . . .    
173	G G . . . . . . . . . . . . . . . . . . . . . . . . . . . . . . .    
174	G G . . . . . . . . . . . . . . . . . . . . . . . . . . . . . . .    
175	G G . . . . . . . . . . . . . . . . . . . . . . . . . . . . . . .    
176	A A . . . . . . . . . . . . . . . . . . . . . . . . . . . . . . .    
177	A A . . . . . . . . . . . . . . . . . . . . . . . . . . . . . . .    
178	G G . . . . . . . . . . . . . . . . . . . . . . . . . . . . . . .    
179	G G . . . . . . . . . . . . . . . . . . . . . . . . . . . . . . .    
180	G G . . . . . . . . . . . . . . . . . . . . . . . . . . . . . . .    
181	G G . . . . . . . . . . . . . . . . . . . . . . . . . . . . . . .    
182	G G . . . . . . . . . . . . . . . . . . . . . . . . . . . . . . .    
183	T T . . . . . . . . . . . . . . . . . . . . . . . . . . . . . . .    
184	G G . . . . . . . . . . . . . . . . . . . . . . . . . . . . . . .    
185	G G . . . . . . . . . . . . . . . . . . . . . . . . . . . . . . .    
186	T T . . . . . . . . . . . . . . . . . . . . . . . . . . . . . . .    
187	T T . . . . . . . . . . . . . . . . . . . . . . . . . . . . . . .    
188	T T . . . . . . . . . . . . . . . . . . . . . . . . . . . . . . .    
189	G G . . . . . . . . . . . . . . . . . . . . . . . . . . . . . . .    
190	G G . . . . . . . . . . . . . . . . . . . . . . . . . . . . . . .    
191	A A . . . . . . . . . . . . . . . . . . . . . . . . . . . . . . .    
192	A A . . . . . . . . . . . . . . . . . . . . . . . . . . . . . . .    
193	T T . . . . . . . . . . . . . . . . . . . . . . . . . . . . . . .    
194	T T . . . . . . . . . . . . . . . . . . . . . . . . . . . . . . .    
195	C C . . . . . . . . . . . . . . . . . . . . . . . . . . . . . . .    
196	A A . . . . . . . . . . . . . . . . . . . . . . . . . . . . . . .    
197	G G . . . . . . . . . . . . . . . . . . . . . . . . . . . . . . .    
198	A A . . . . . . . . . . . . . . . . . . . . . . . . . . . . . . .    
199	G G . . . . . . . . . . . . . . . . . . . . . . . . . . . . . . .    
200	T T . . . . . . . . . . . . . . . . . . . . . . . . . . . . . . .    
201	T T . . . . . . . . . . . . . . . . . . . . . . . . . . . . . . .    
202	T T . . . . . . . . . . . . . . . . . . . . . . . . . . . . . . .    
203	C C . . . . . . . . . . . . . . . . . . . . . . . . . . . . . . .    
204	T T . . . . . . . . . . . . . . . . . . . . . . . . . . . . . . .    
205	C C . . . . . . . . . . . . . . . . . . . . . . . . . . . . . . .    
206	T T . . . . . . . . . . . . . . . . . . . . . . . . . . . . . . .    
207	C C . . . . . . . . . . . . . . . . . . . . . . . . . . . . . . .    
208	A A . . . . . . . . . . . . . . . . . . . . . . . . . . . . . . .    
209	T T . . . . . . . . . . . . . . . . . . . . . . . . . . . . . . .    
210	A A . . . . . . . . . . . . . . . . . . . . . . . . . . . . . . .    
211	T T . . . . . . . . . . . . . . . . . . . . . . . . . . . . . . .    
212	G G . . . . . . . . . . . . . . . . . . . . . . . . . . . . . . .    
213	C C . . . . . . . . . . . . . . . . . . . . . . . . . . . . . . .    
214	T T . . . . . . . . . . . . . . . . . . . . . . . . . . . . . . .    
215	T T . . . . . . . . . . . . . . . . . . . . . . . . . . . . . . .    
216	C C . . . . . . . . . . . . . . . . . . . . . . . . . . . . . . .    
217	C C . . . . . . . . . . . . . . . . . . . . . . . . . . . . . . .    
218	A A . . . . . . . . . . . . . . . . . . . . . . . . . . . . . . .    
219	A A . . . . . . . . . . . . . . . . . . . . . . . . . . . . . . .    
220	T T . . . . . . . . . . . . . . . . . . . . . . . . . . . . . . .    
221	T T . . . . . . . . . . . . . . . . . . . . . . . . . . . . . . .    
222	G G . . . . . . . . . . . . . . . . . . . . . . . . . . . . . . .    
223	A A . . . . . . . . . . . . . . . . . . . . . . . . . . . . . . .    
224	A A . . . . . . . . . . . . . . . . . . . . . . . . . . . . . . .    
225	A A . . . . . . . . . . . . . . . . . . . . . . . . . . . . . . .    
226	T T . . . . . . . . . . . . . . . . . . . . . . . . . . . . . . .    
227	G G . . . . . . . . . . . . . . . . . . . . . . . . . . . . . . .    
228	T T . . . . . . . . . . . . . . . . . . . . . . . . . . . . . . .    
229	A A . . . . . . . . . . . . . . . . . . . . . . . . . . . . . . .    
230	T T . . . . . . . . . . . . . . . . . . . . . . . . . . . . . . .    
231	G G . . . . . . . . . . . . . . . . . . . . . . . . . . . . . . .    
232	G G . . . . . . . . . . . . . . . . . . . . . . . . . . . . . . .    
233	C C . . . . . . . . . . . . . . . . . . . . . . . . . . . . . . .    
234	T T . . . . . . . . . . . . . . . . . . . . . . . . . . . . . . .    
235	A A . . . . . . . . . . . . . . . . . . . . . . . . . . . . . . .    
236	T T . . . . . . . . . . . . . . . . . . . . . . . . . . . . . . .    
237	A A . . . . . . . . . . . . . . . . . . . . . . . . . . . . . . .    
238	G G . . . . . . . . . . . . . . . . . . . . . . . . . . . . . . .    
239	G G . . . . . . . . . . . . . . . . . . . . . . . . . . . . . . .    
240	G G . . . . . . . . . . . . . . . . . . . . . . . . . . . . . . .    
241	A A . . . . . . . . . . . . . . . . . . . . . . . . . . . . . . .    
242	T T . . . . . . . . . . . . . . . . . . . . . . . . . . . . . . .    
243	G G . . . . . . . . . . . . . . . . . . . . . . . . . . . . . . .    
244	G G . . . . . . . . . . . . . . . . . . . . . . . . . . . . . . .    
245	A A . . . . . . . . . . . . . . . . . . . . . . . . . . . . . . .    
246	T T . . . . . . . . . . . . . . . . . . . . . . . . . . . . . . .    
247	C C . . . . . . . . . . . . . . . . . . . . . . . . . . . . . . .    
248	T T . . . . . . . . . . . . . . . . . . . . . . . . . . . . . . .    
249	C C . . . . . . . . . . . . . . . . . . . . . . . . . . . . . . .    
250	T T . . . . . . . . . . . . . . . . . . . . . . . . . . . . . . .    
251	G G . . . . . . . . . . . . . . . . . . . . . . . . . . . . . . .    
252	T T . . . . . . . . . . . . . . . . . . . . . . . . . . . . . . .    
253	G G . . . . . . . . . . . . . . . . . . . . . . . . . . . . . . .    
254	T T . . . . . . . . . . . . . . . . . . . . . . . . . . . . . . .    
255	G G . . . . . . . . . . . . . . . . . . . . . . . . . . . . . . .    
256	T T . . . . . . . . . . . . . . . . . . . . . . . . . . . . . . .    
257	A A . . . . . . . . . . . . . . . . . . . . . . . . . . . . . . .    
258	A A . . . . . . . . . . . . . . . . . . . . . . . . . . . . . . .    
259	T T . . . . . . . . . . . . . . . . . . . . . . . . . . . . . . .    
260	T T . . . . . . . . . . . . . . . . . . . . . . . . . . . . . . .    
261	G G . . . . . . . . . . . . . . . . . . . . . . . . . . . . . . .    
262	C C . . . . . . . . . . . . . . . . . . . . . . . . . . . . . . .    
263	A A . . . . . . . . . . . . . . . . . . . . . . . . . . . . . . .    
264	T T . . . . . . . . . . . . . . . . . . . . . . . . . . . . . . .    
265	G G . . . . . . . . . . . . . . . . . . . . . . . . . . . . . . .    
266	A A . . . . . . . . . . . . . . . . . . . . . . . . . . . . . . .    
267	G G . . . . . . . . . . . . . . . . . . . . . . . . . . . . . . .    
268	T T . . . . . . . . . . . . . . . . . . . . . . . . . . . . . . .    
269	G G . . . . . . . . . . . . . . . . . . . . . . . . . . . . . . .    
270	A A . . . . . . . . . . . . . . . . . . . . . . . . . . . . . . .    
271	G G . . . . . . . . . . . . . . . . . . . . . . . . . . . . . . .    
272	C C . . . . . . . . . . . . . . . . . . . . . . . . . . . . . . .    
273	A A . . . . . . . . . . . . . . . . . . . . . . . . . . . . . . .    
274	G G . . . . . . . . . . . . . . . . . . . . . . . . . . . . . . .    
275	G G . . . . . . . . . . . . . . . . . . . . . . . . . . . . . . .    
276	T T . . . . . . . . . . . . . . . . . . . . . . . . . . . . . . .    
277	T T . . . . . . . . . . . . . . . . . . . . . . . . . . . . . . .    
278	A A . . . . . . . . . . . . . . . . . . . . . . . . . . . . . . .    
279	T T . . . . . . . . . . . . . . . . . . . . . . . . . . . . . . .    
280	A A . . . . . . . . . . . . . . . . . . . . . . . . . . . . . . .    
281	C C . . . . . . . . . . . . . . . . . . . . . . . . . . . . . . .    
282	C C . . . . . . . . . . . . . . . . . . . . . . . . . . . . . . .    
283	C C . . . . . . . . . . . . . . . . . . . . . . . . . . . . . . .    
284	T T . . . . . . . . . . . . . . . . . . . . . . . . . . . . . . .    
285	T T . . . . . . . . . . . . . . . . . . . . . . . . . . . . . . .    
286	T T . . . . . . . . . . . . . . . . . . . . . . . . . . . . . . .    
287	T T . . . . . . . . . . . . . . . . . . . . . . . . . . . . . . .    
288	A A . . . . . . . . . . . . . . . . . . . . . . . . . . . . . . .    
289	G G . . . . . . . . . . . . . . . . . . . . . . . . . . . . . . .    
290	G G . . . . . . . . . . . . . . . . . . . . . . . . . . . . . . .    
291	A A . . . . . . . . . . . . . . . . . . . . . . . . . . . . . . .    
292	A A . . . . . . . . . . . . . . . . . . . . . . . . . . . . . . .    
293	G G . . . . . . . . . . . . . . . . . . . . . . . . . . . . . . .    
294	C C . . . . . . . . . . . . . . . . . . . . . . . . . . . . . . .    
295	T T . . . . . . . . . . . . . . . . . . . . . . . . . . . . . . .    
296	C C . . . . . . . . . . . . . . . . . . . . . . . . . . . . . . .    
297	T T . . . . . . . . . . . . . . . . . . . . . . . . . . . . . . .    
298	C C . . . . . . . . . . . . . . . . . . . . . . . . . . . . . . .    
299	T T . . . . . . . . . . . . . . . . . . . . . . . . . . . . . . .    
300	T T . . . . . . . . . . . . . . . . . . . . . . . . . . . . . . .    
301	G G . . . . . . . . . . . . . . . . . . . . . . . . . . . . . . .    
302	T T . . . . . . . . . . . . . . . . . . . . . . . . . . . . . . .    
303	T T . . . . . . . . . . . . . . . . . . . . . . . . . . . . . . .    
304	G G . . . . . . . . . . . . . . . . . . . . . . . . . . . . . . .    
305	T T . . . . . . . . . . . . . . . . . . . . . . . . . . . . . . .    
306	G G . . . . . . . . . . . . . . . . . . . . . . . . . . . . . . .    
307	A A . . . . . . . . . . . . . . . . . . . . . . . . . . . . . . .    
308	A A . . . . . . . . . . . . . . . . . . . . . . . . . . . . . . .    
309	A A . . . . . . . . . . . . . . . . . . . . . . . . . . . . . . .    
310	T T . . . . . . . . . . . . . . . . . . . . . . . . . . . . . . .    
311	A A . . . . . . . . . . . . . . . . . . . . . . . . . . . . . . .    
312	T T . . . . . . . . . . . . . . . . . . . . . . . . . . . . . . .    
313	A A . . . . . . . . . . . . . . . . . . . . . . . . . . . . . . .    
314	A A . . . . . . . . . . . . . . . . . . . . . . . . . . . . . . .    
315	T T . . . . . . . . . . . . . . . . . . . . . . . . . . . . . . .    
316	T T . . . . . . . . . . . . . . . . . . . . . . . . . . . . . . .    
317	A A . . . . . . . . . . . . . . . . . . . . . . . . . . . . . . .    
318	T T . . . . . . . . . . . . . . . . . . . . . . . . . . . . . . .    
319	T T . . . . . . . . . . . . . . . . . . . . . . . . . . . . . . .    
320	T T . . . . . . . . . . . . . . . . . . . . . . . . . . . . . . .    
321	T T . . . . . . . . . . . . . . . . . . . . . . . . . . . . . . .    
322	A A . . . . . . . . . . . . . . . . . . . . . . . . . . . . . . .    
323	A A . . . . . . . . . . . . . . . . . . . . . . . . . . . . . . .    
324	G G . . . . . . . . . . . . . . . . . . . . . . . . . . . . . . .    
325	A A . . . . . . . . . . . . . . . . . . . . . . . . . . . . . . .    
326	A A . . . . . . . . . . . . . . . . . . . . . . . . . . . . . . .    
327	G G G . . G . . . . . . . . . . . . . . . . . . . . . . . . . . .    
328	G G G . . G . . . . . . . . . . . . . . . . . . . . . . . . . . .    
329	A A A . . A . . . . . . . . . . . . . . . . . . . . . . . . . . .    
330	C C C . . C . . . . . . . . . . . . . . . . . . . . . . . . . . .    
331	T T T . . T . . . . . . . . . . . . . . . . . . . . . . . . . . .    
332	G G G . . G . . . . . . . . . . . . . . . . . . . . . . . . . . .    
333	C C C . . C . . . . . . . . . . . . . . . . . . . . . . . . . . .    
334	C C C . . C . . . . . . . . . . . . . . . . . . . . . . . . . . .    
335	T T T . . T . . . . . . . . . . . . . . . . . . . . . . . . . . .    
336	T T T . . T . . . . . . . . . . . . . . . . . . . . . . . . . . .    
337	C C C . . C . . . . . . . . . . . . . . . . . . . . . . . . . . .    
338	G G G . . G . . . . . . . . . . . . . . . . . . . . . . . . . . .    
339	T T T . . T . . . . . . . . . . . . . . . . . . . . . . . . . . .    
340	T T T . . T . . . . . . . . . . . . . . . . . . . . . . . . . . .    
341	G G G . . G . . . . . . . . . . . . . . . . . . . . . . . . . . .    
342	T T T . . T . . . . . . . . . . . . . . . . . . . . . . . . . . .    
343	T T T . . T . . . . . . . . . . . . . . . . . . . . . . . . . . .    
344	T T T . . T . . . . . . . . . . . . . . . . . . . . . . . . . . .    
345	G G G . . G . . . . . . . . . . . . . . . . . . . . . . . . . . .    
346	G G G . . G . . G . . . . . . . . . . . . . . . . . . . . . . . .    
347	G G G . . G . . G . . . . . . . . . . . . . . . . . . . . . . . .    
348	A A A . . A . . A . . . . . . . . . . . . . . . . . . . . . . . .    
349	G G G . . G . . G . . . . . . . . . . . . . . . . . . . . . . . .    
350	T T T . . T . . T . . . . . . . . . . . . . . . . . . . . . . . .    
351	T T T . . T . . T . . . . . . . . . . . . . . . . . . . . . . . .    
352	C C C . . C . . C . . . . . . . . . . . . . . . . . . . . . . . .    
353	A A A . . A . . A . . . . . . . . . . . . . . . . . . . . . . . .    
354	T T T . . T . . T . . . . . . . . . . . . . . . . . . . . . . . .    
355	T T T . . T . . T . . . . . . . . . . . . . . . . . . . . . . . .    
356	T T T . . T . . T . . . . . . . . . . . . . . . . . . . . . . . .    
357	G G G . . G . . G . . . . . . . . . . . . . . . . . . . . . . . .    
358	G G G . . G . . G . . . . . . . . . . . . . . . . . . . . . . . .    
359	C C C . . C . . C . . . . . . . . . . . . . . . . . . . . . . . .    
360	T T T . . T . . T . . . . . . . . . . . . . . . . . . . . . . . .    
361	T T T . . T . . T . . . . . . . . . . . . . . . . . . . . . . . .    
362	T T T . . T . . T . . . . . . . . . . . . . . . . . . . . . . . .    
363	T T T . . T . . T . . . . . . . . . . . . . . . . . . . . . . . .    
364	G G G . . G . . G . . . . . . . . . . . . . . . . . . . . . . . .    
365	T T T . . T . . T . . . . . . . . . . . . . . . . . . . . . . . .    
366	A A A . . A . . A . . . . . . . . . . . . . . . . . . . . . . . .    
367	G G G . . G G G G . . G . . . . . . . . . . . . . . . . . . . . .    
368	A A A . . A A A A . . A . . . . . . . . . . . . . . . . . . . . .    
369	T T T . . T T T T . . T . . . . . . . . . . . . . . . . . . . . .    
370	A A A . . A A A A . . A A . . . . . . . . . . . . . . . . . . . .    
371	T T T . . T T T T . . T T . . . . . . . . . . . . . . . . . . . .    
372	A A A . . A A A A . . A A . . . . . . . . . . . . . . . . . . . .    
373	A A A . . A A A A . . A A . . . . . . . . . . . . . . . . . . . .    
374	G G G . . G G G G . . G G . . . . . . . . . . . . . . . . . . . .    
375	A A A . . A A A A . . A A . . . . . . . . . . . . . . . . . . . .    
376	A A A . . A A A A . . A A . . . . . . . . . . . . . . . . . . . .    
377	A A A . . A A A A . . A A . . . . . . . . . . . . . . . . . . . .    
378	T T T . . T T T T . . T T . . . . . . . . . . . . . . . . . . . .    
379	A A A . . A A A A . . A A . . . . . . . . . . . . . . . . . . . .    
380	A A A . . A A A A . . A A . . . . . . . . . . . . . . . . . . . .    
381	T T T . . T T T T . . T T . . . . . . . . . . . . . . . . . . . .    
382	T T T . . T T T T . . T T . . . . . . . . . . . . . . . . . . . .    
383	G G G . . G G G G . . G G . . . . . . . . . . . . . . . . . . . .    
384	C C C . . C C C C . . C C . . . . . . . . . . . . . . . . . . . .    
385	C C C . . C C C C . . C C . . . . . . . . . . . . . . . . . . . .    
386	A A A . . A A A A . . A A . . . . . . . . . . . . . . . . . . . .    
387	T T T . . T T T T . . T T . . . . . . . . . . . . . . . . . . . .    
388	C C C . . C C C C . . C C . . . . . . . . . . . . . . . . . . . .    
389	A A A A . A A A A . . A A . . . . . . . . . . . . . . . . . . . .    
390	C C C C . C C C C . . C C . . . . . . . . . . . . . . . . . . . .    
391	T T T T . T T T T . . T T . . . . . . . . . . . . . . . . . . . .    
392	C C C C . C C C C . . C C . . . . . . . . . . . . . . . . . . . .    
393	T T T T . T T T T . . T T . . . . . . . . . . . . . . . . . . . .    
394	C C C C . C C C C . . C C . . . . . . . . . . . . . . . . . . . .    
395	C C C C . C C C C . . C C . . . . . . . . . . . . . . . . . . . .    
396	C C C C . C C C C . . C C . . . . . . . . . . . . . . . . . . . .    
397	A A A A . A A A A . . A A . . . . . . . . . . . . . . . . . . . .    
398	T T T T . T T T T . . T T . . . . . . . . . . . . . . . . . . . .    
399	G G G G . G G G G . . G G . . . . . . . . . . . . . . . . . . . .    
400	T T T T . T T T T . . T T . . . . . . . . . . . . . . . . . . . .    
401	G G G G . G G G G . . G G . . . . . . . . . . . . . . . . . . . .    
402	A A A A . A A A A . A A A . . . . . . . . . . . . . . . . . . . .    
403	G G G G . G G G G . G G G . . . . . . . . . . . . . . . . . . . .    
404	C C C C . C C C C . C C C . . . . . . . . . . . . . . . . . . . .    
405	A A A A . A A A A . A A A . . . . . . . . . . . . . . . . . . . .    
406	G G G G . G G G G . G G G . . . . . . . . . . . . . . . . . . . .    
407	T T T T . T T T T . T T T . . . . . . . . . . . . . . . . . . . .    
408	C C C C . C C C C . C C C . . . . . . . . . . . . . . . . . . . .    
409	A A A A . A A A A . A A A . . . . . . . . . . . . . . . . . . . .    
410	T T T T . T T T T . T T T . . . . . . . . . . . . . . . . . . . .    
411	A A A A . A A A A . A A A . . . . . . . . . . . . . . . . . . . .    
412	T T T T . T T T T . T T T . . . . . . . . . . . . . . . . . . . .    
413	G G G G . G G G G . G G G . . . . . . . . . . . . . . . . . . . .    
414	C C C C . C C C C . C C C . . . . . . . . . . . . . . . . . . . .    
415	A A A A . A A A A . A A A . . . . . . . . . . . . . . . . . . . .    
416	G G G G . G G G G . G G G . . . . . . . . . . . . . . . . . . . .    
417	T T T T . T T T T . T T T . . . . . . . . . . . . . . . . . . . .    
418	G G G G . G G G G . G G G . . . . . . . . . . . . . . . . . . . .    
419	T T T T . T T T T . T T T . . . . . . . . . . . . . . . . . . . .    
420	T T T T . T T T T . T T T . . . . . . . . . . . . . . . . . . . .    
421	G G G G . G G G G . G G G . . . . . . . . . . . . . . . . . . . .    
422	T T T T . T T T T . T T T . . . . . . . . . . . . . . . . . . . .    
423	A A A A . A A A A . A A A . . . . . . . . . . . . . . . . . . . .    
424	T T T T . T T T T . T T T . . . . . . . . . . . . . . . . . . . .    
425	T T T T . T T T T . T T T . . . . . . . . . . . . . . . . . . . .    
426	T T T T . T T T T . T T T . . . . . . . . . . . . . . . . . . . .    
427	T T T T . T T T T . T T T . . . . . . . . . . . . . . . . . . . .    
428	T T T T . T T T T . T T T . . . . . . . . . . . . . . . . . . . .    
429	A A A A . A A A A . A A A . . . . . . . . . . . . . . . . . . . .    
430	C C C C . C C C C . C C C . . . . . . . . . . . . . . . . . . . .    
431	C C C C . C C C C . C C C . . . . . . . . . . . . . . . . . . . .    
432	A A A A . A A A A . A A A . . . . . . . . . . . . . . . . . . . .    
433	C C C C . C C C C . C C C . . . . . . . . . . . . . . . . . . . .    
434	A A A A . A A A A . A A A . . . . . . . . . . . . . . . . . . . .    
435	. A A A . A A A A . A A A . . . . . . . . . . . . . . . . . . . .    
436	. G G G . G G G G . G G G . . . . . . . . G . . . . . . . . . . .    
437	. . A A . A A A A . A A A . . . . . . . . A . . . . . . . . . . .    
438	. . G G . G G G G . G G G . . . . . . . . G . . . . . . . . . . .    
439	. . G G . G G G G . G G G . . . . . . . . G . . . . . . . . . . .    
440	. . G G . G G G G . G G G . . . . . . . . G . . . . . . . . . . .    
441	. . G G . G G G G . G G G . . . . . . . . G . . . . . . . . . . .    
442	. . G G . G G G G . G G G . . . . . . . . G . . . . . . . . . . .    
443	. . A A . A A A A . A A A . . . . . . . . A . . . . . . . . . . .    
444	. . G G . G G G G . G G G . . . . . . . . G . . . . . . . . . . .    
445	. . G G . G G G G . G G G . . . . . . . . G . . . . . . . . . . .    
446	. . T T . T T T T . T T T . . . . . . . . T . . . . . . . . . . .    
447	. . G G . G G G G . G G G . . . . . . . . G . G . . . . . . . . .    
448	. . T T . T T T T . T T T . . . . . . . . T . T . . . . . . . . .    
449	. . G G . G G G G . G G G . . . . . . . . G . G . . . . . . . . .    
450	. . A A . A A A A . A A A . . . . . . . . A . A . . . . . . . . .    
451	. . G G . G G G G . G G G . . . . . . . . G . G . . . . . . . . .    
452	. . G G . G G G G . G G G . . . . . . . . G . G . . . . . . . . .    
453	. . A A . A A A A . A A A . . . . . . . . A . A . . . . . . . . .    
454	. . T T . T T T T . T T T . . . . . . . . T . T . . . . . . . . .    
455	. . T T . T T T T . T T T . . . . . . . . T . T . . . . . . . . .    
456	. . G G . G G G G . G G G . . . . . . . . G . G . . . . . . . . .    
457	. . G G . G G G G . G G G . . . . . . . . G . G . . . . . . . . .    
458	. . T T . T T T T . T T T . . . . . . . . T . T . . . . . . . . .    
459	. . A A . A A A A . A A A . . . . . . . . A . A . . . . . . . . .    
460	. . C C . C C C C . C C C . . . . . . . . C . C . . . . . . . . .    
461	. . A A . A A A A . A A A . . . . . . . . A . A . . . . . . . . .    
462	. . G G . G G G G . G G G . . . . . . . . G . G . . . . . . . . .    
463	. . A A . A A A A . A A A . . . . . . . . A . A . . . . . . . . .    
464	. . C C . C C C C . C C C . . . . . . . . C . C . . . . . . . . .    
465	. . A A . A A A A . A A A . . . . . . . . A . A . . . . . . . . .    
466	. . G G . G G G G . G G G . . . . . . . . G . G . . . . . . . . .    
467	. . A A . A A A A . A A A . . . . . . . . A . A . . . . . . . . .    
468	. . A A . A A A A . A A A . . . . . . . . A . A . . . . . . . . .    
469	. . G G . G G G G . G G G . . . . . . . . G . G . . . . . . . . .    
470	. . C C . C C C C . C C C . . . . C . . . C . C . . . . . . . . .    
471	. . A A . A A A A . A A A . . . . A . . . A . A . . . . . . . . .    
472	. . A A . A A A A . A A A . . . . A . . . A . A . . . . . . . . .    
473	. . C C . C C C C . C C C . . . . C . . . C . C . . . . . . . . .    
474	. . T T . T T T T . T T T . . . . T . . . T . T . . . . . . . . .    
475	. . G G . G G G G . G G G . . . . G . . . G . G . . . . . . . . .    
476	. . G G . G G G G . G G G . . . . G . . . G . G . . . . . . . . .    
477	. . C C . C C C C . C C C . . . . C . . . C . C . . . . . . . . .    
478	. . T T . T T T T . T T T . . . . T . . . T . T . . . . . . . . .    
479	. . T T . T T T T . T T T . . . . T . . . T . T . . . . . . . . .    
480	. . G G . G G G G . G G G . . . . G . . . G . G . . . . . . . . .    
481	. . A A . A A A A . A A A . . . . A . . . A . A . . . . . . . . .    
482	. . A A . A A A A . A A A . . . . A . . . A . A . . . . . . . . .    
483	. . G G . G G G G . G G G . . . . G . . . G . G . . . . . . . . .    
484	. . G G . G G G G . G G G . . . . G . . . G . G . . . . . . . . .    
485	. . G G . G G G G . G G G . . . . G . . . G . G . . . . . . . . .    
486	. . T T . T T T T . T T T . . . . T . . . T . T . . . . . . . . .    
487	. . A A . A A A A . A A A . . . . A . . . A . A . . . . . . . . .    
488	. . G G . G G G G . G G G . . . . G . . . G . G . . . . . . . . .    
489	. . T T . T T T T . T T T . . . . T . . . T . T . . . . . . . . .    
490	. . T T . T T T T . T T T . . . . T . . . T . T . . . . . . . . .    
491	. . T T . T T T T . T T T . . . . T . . . T . T . . . . . . . . .    
492	. . G G . G G G G . G G G . . . . G . . . G . G . . . . . . . . .    
493	. . A A . A A A A . A A A . . . . A . . . A . A . . . . . . . . .    
494	. . C C . C C C C . C C C . . . . C . . . C . C . . . . . . . . .    
495	. . C C . C C C C . C C C . . . . C . . . C . C . . . . . . . . .    
496	. . A A . A A A A . A A A . . . . A . . . A . A . . . . . . . . .    
497	. . A A . A A A A . A A A . . . . A . . . A . A . . . . . . . . .    
498	. . A A . A A A A . A A A . . . . A . . . A . A . . . . . . . . .    
499	. . T T . T T T T . T T T . . . . T . . . T . T . . . . . . . . .    
500	. . T T . T T T T . T T T . . . . T . . . T . T . . . . . . . . .    
501	. . A A . A A A A . A A A . . . . A . . . A . A . . . . . . . . .    
502	. . C C . C C C C . C C C . . . . C . . . C . C . . . . . . . . .    
503	. . A A . A A A A . A A A . . . . A . . . A . A . . . . . . . . .    
504	. . T T . T T T T . T T T . . . . T . . . T . T . . . . . . . . .    
505	. . C C . C C C C . C C C . . . . C . . . C . C . . . . . . . . .    
506	. . T T . T T T T . T T T . . . . T . . . T . T . . . . . . . . .    
507	. . G G . G G G G . G G G . . . . G . . . G . G . . . . . . . . .    
508	. . T T . T T T T . T T T . . . . T . . . T . T . . . . . . . . .    
509	. . T T . T T T T . T T T . . . . T . . . T . T . . . T . . . . .    
510	. . G G . G G G G . G G G . . . . G . . . G . G . . . G . . G G .    
511	. . T T . T T T T . T T T . . . . T . . . T . T . . . T . . T T .    
512	. . T T . T T T T . T T T . . . . T . . . T . T . . . T . . T T .    
513	. . G G . G G G G . G G G . . . . G . . . G . G . . . G . . G G .    
514	. . T T . T T T T . T T T . . . . T . . . T . T . . . T . . T T .    
515	. . A A . A A A A . A A A . . . . A . . . A . A . . . A . . A A .    
516	. . G G . G G G G . G G G . . . . G . . . G . G . . . G . . G G .    
517	. . T T . T T T T . T T T . . . . T . . . T . T . . . T . . T T .    
518	. . C C . C C C C . C C C . . . . C . . . C . C . . . C . . C C .    
519	. . A A . A A A A . A A A . . . . A . . . A . A . . . A . . A A .    
520	. . G G . G G G G . G G G . . . . G . . . G . G . . . G . . G G .    
521	. . G G . G G G G . G G G . . . . G . . . G . G . . . G . . G G .    
522	. . A A . A A A A . A A A . . . . A . . . A . A . . . A . . A A .    
523	. . G G . G G G G . G G G . . . . G . . . G . G . . . G . . G G .    
524	. . G G . G G G G . G G G . . . . G . . . G . G . . . G . . G G .    
525	. . T T . T T T T . T T T . . . . T . . . T . T . . . T . . T T .    
526	. . G G . G G G G . G G G . . . . G . . . G . G . . . G . G G G .    
527	. . A A . A A A A . A A A . . . . A . . . A . A . . . A . A A A .    
528	. . T T . T T T T . T T T . . . . T . . . T . T . . . T . T T T .    
529	. . T T . T T T T . T T T . . . . T . . . T . T . . . T . T T T .    
530	. . G G . G G G G . G G G . . . . G . . . G . G . . . G . G G G .    
531	. . T T . T T T T . T T T . . . . T . . . T . T . . . T . T T T .    
532	. . T T . T T T T . T T T . . . . T . . . T . T . . . T . T T T .    
533	. . T T . T T T T . T T T . . . . T . . . T . T . . . T . T T T .    
534	. . T T . T T T T . T T T . . . . T . . . T . T . . . T . T T T .    
535	. . T T . T T T T . T T T . . . . T . . . T . T . . . T . T T T .    
536	. . G G . G G G G . G G G . . . . G . . . G . G . . . G . G G G .    
537	. . T T . T T T T . T T T . . . . T . . . T . T . . . T . T T T .    
538	. . A A . A A A A . A A A . . . . A . . . A . A . . . A . A A A .    
539	. . A A . A A A A . A A A . . . . A . . . A . A . . . A . A A A .    
540	. . T T . T T T T . T T T . . . . T . . . T . T . . . T . T T T .    
541	. . G G . G G G G . G G G . . . . G . . . G . G . . . G . G G G .    
542	. . C C . C C C C . C C C . . . . C . . . C . C . . . C . C C C .    
543	. . C C . C C C C . C C C . . . . C . . . C . C . . . C . C C C .    
544	. . A A . A A A A . A A A . . . . A . . . A . A . . . A . A A A .    
545	. . T T . T T T T . T T T . . . . T . . . T . T . . . T . T T T .    
546	. . T T . T T T T . T T T . . . . N . . . T . T . . . T . T T T .    
547	. . T T . T T T T . T T T . . . . T . . . T . T . . . T . T T T .    
548	. . G G . G G G G . G G G . . . . G . . . G . G . G G G . G G G .    
549	. . G G . G G G G . G G G . . . . G . . . G . G . G G G . G G G .    
550	. . G G . G G G G . G G G . . . . G . . . G . G . G G G . G G G .    
551	. . G G . G G G G . G G G . . . . G . . . G . G . G G G . G G G .    
552	. . T T . T T T T . T T T . . . . T . . . T . T . T T T . T T T .    
553	. . A A . A A A A . A A A . . . . A . . . A . A . A A A . A A A .    
554	. . T T . T T T T . T T T . . . . T . . . T . T . T T T . T T T .    
555	. . G G . G G G G . G G G . . . . G . . . G . G . G G G . G G G .    
556	. . C C . C C C C . C C C . . . . C . . . C . C . C C C . C C C .    
557	. . C C . C C C C . C C C . . . . C . . . C . C . C C C . C C C .    
558	. . A A . A A A A . A A A . . . . A . . . A . A . A A A . A A A .    
559	. . A A . A A A A . A A A . . . . A . . . A . A . A A A . A A A .    
560	. . C C . C C C C . C C C . . . . C . . . C . C . C C C . C C C .    
561	. . A A . A A A A . A A A . . . . A . . . A . A . A A A . A A A .    
562	. . A A . A A A A . A A A . . . . A . . . A . A . A A A . A A A .    
563	. . G G . G G G G . G G G . . . . G . . . G . G . G G G . G G G .    
564	. . G G . G G G G . G G G . . . . G . . . G . G . G G G . G G G .    
565	. . G G . G G G G . G G G . . . . G . . . G . G . G G G . G G G .    
566	. . T T . T T T T . T T T . . . . T . . . T . T . T T T . T T T .    
567	. . A A A A A A A . A A A . . . . A . . . A . A . A A A . A A A .    
568	. . C C C C C C C . C C C . . . . C . . . C . C . C C C . C C C .    
569	. . T T T T T T T . T T T . . . . T . . . T . T . T T T . T T T .    
570	. . A A A A A A A . A A A . . . . A . . . A . A . A A A . A A A .    
571	. . C C C C C C C . C C C . . . . C . . . C . C . C C C . C C C .    
572	. . C C C C C C C . C C C . . . . C . . . C . C . C C C . C C C .    
573	. . A A A A A A A . A A A . . . . A . . . A . A . A A A . A A A .    
574	. . C C C C C C C . C C C . . C . C . . . C . C . C C C . C C C .    
575	. . T T T T T T T . T T T . . T . T . . . T . T . T T T . T T T .    
576	. . C C C C C C C . C C C . . C . C . . . C . C . C C C . C C C .    
577	. . C C C C C C C . C C C . . C . C . . . C . C . C C C . C C C .    
578	. . A A A A A A A . A A A . . A . A . . . A . A . A A A . A A A .    
579	. . T T T T T T T . T T T . . T . T . . . T . T . T T T . T T T .    
580	. . T T T T T T T . T T T . . T . T . . . T . T . T T T . T T T .    
581	. . T T T T T T T . T T T . . T . T . . . T . T . T T T . T T T .    
582	. . C C C C C C C . C C C . . C . C . . . C . C . C C C . C C C .    
583	. . T T T T T T T . T T T . . T . T . . . T . T . T T T . T T T .    
584	. . T T T T T T T . T T T . . T T T . . . T . T . T T T . T T T .    
585	. . T T T T T T T . T T T . . T T T . . . T . T . T T T . T T T .    
586	. . T T T T T T T . T T T . . T T T . . . T . T . T T T . T T T .    
587	. . G G G G G G G . G G G . . G G G . . . G . G . G G G . G G G .    
588	. . G G G G G G G . G G G . . G G G . . . G . G . G G G . G G G .    
589	. . A A A A A A A A A A A . . A A A A . . A . A . A A A . A A A .    
590	. . C C C C C C C C C C C . . C C C C . . C . C . C C C . C C C .    
591	. . A A A A A A A A A A A . . A A A A . . A . A . A A A . A A A .    
592	. . A A A A A A A A A A A . . A A A A . . A . A . A A A . A A A .    
593	. . T T T T T T T T T T T . . T T T T . . T . T . T T T . T T T .    
594	. . G G G G G G G G G G G . . G G G G . . G . G . G G G . G G G .    
595	. . C C C C C C C C C C C . . C C C C . . C . C . C C C . C C C .    
596	. . C C C C C C C C C C C . . C C C C . . C . C . C C C . C C C .    
597	. . A A A A A A A A A A A . . A A A A . . A . A . A A A . A A A .    
598	. . C C C C C C C C C C C . . C C C C . . C . C . C C C . C C C .    
599	. . C C C C C C C C C C C . . C C C C . . C . C . C C C . C C C C    
600	. . A A A A A A A A A A A . . A A A A . . A . A . A A A . A A A A    
601	. . A A A A A A A A A A A . . A A A A . . A . A . A A A . A A A A    
602	. . T T T T T T T T T T T . . T T T T . . T . T . T T T . T T T T    
603	. . A A A A A A A A A A A . . A A A A . . A . A . A A A . A A A A    
604	. . A A A A A A A A A A A . . A A A A . A A . A . A A A . A A A A    
605	. . G G G G G G G G G G G G . G G G G . G G . G . G G G . G G G G    
606	. . C C C C C C C C C C C C . C C C C . C C . C . C C C . C C C C    
607	. . T T T T T T T T T T T T . T T T T . T T . T . T T T . T T T T    
608	. . G G G G G G G G G G G G . G G G G . G G . G . G G G . G G G G    
609	. . T T T T T T T T T T T T . T T T T . T T . T . T T T . T T T T    
610	. . A A A A A A A A A A A A . A A A A . A A . A . A A A . A A A A    
611	. . G G G G G G G G G G G G . G G G G . G G . G . G G G . G G G G    
612	. . C C C C C C C C C C C C . C C C C . C C . C . C C C . C C C C    
613	. . A A A A A A A A A A A A . A A A A . A A . A . A A A . A A A A    
614	. . A A A A A A A A A A A A . A A A A . A A . A . A A A . A A A A    
615	. . G G G G G G G G G G G G . G G G G . G G . G . G G G . G G G G    
616	. . G G G G G G G G G G G G . G G G G . G G . G . G G G . G G G G    
617	. . T T T T T T T T T T T T . T T T T . T T . T . T T T . T T T T    
618	. . A A A A A A A A A A A A . A A A A . A A . A . A A A . A A A A    
619	. . A A A A A A A A A A A A . A A A A . A A . A . A A A . A A A A    
620	. . A A A A A A A A A A A A . A A A A . A A . A . A A A . A A A A    
621	. . C C C C C C C C C C C C . C C C C . C C . C . C C C . C C C C    
622	. . A A A A A A A A A A A A . A A A A . A A . A . A A A . A A A A    
623	. . A A A A A A A A A A A A . A A A A . A A . A . A A A . A A A A    
624	. . A A A A A A A A A A A A . A A A A . A A . A . A A A . A A A A    
625	. . C C C C C C C C C C C C . C C C C . C C . C . C C C . C C C C    
626	. . C C C C C C C C C C C C . C C C C . C C . C . C C C . C C C C    
627	. . C C C C C C C C C C C C . C C C C . C C . C . C C C . C C C C    
628	. . A A A A A A A A A A A A . A A A A . A A . A . A A A . A A A A    
629	. . C C C C C C C C C C C C . C C C C . C C . C . C C C . C C C C    
630	. . A A A A A A A A A A A A . A A A A . A A . A . A A A . A A A A    
631	. . T T T T T T T T T T T T . T T T T . T T . T . T T T . T T T T    
632	. . T T T T T T T T T T T T . T T T T . T T . T . T T T . T T T T    
633	. . C C C C C C C C C C C C . C C C C . C C . C . C C C . C C C C    
634	. . T T T T T T T T T T T T . T T T T . T T . T . T T T . T T T T    
635	. . G G G G G G G G G G G G . G G G G . G G . G . G G G . G G G G    
636	. . G G G G G G G G G G G G . G G G G . G G . G . G G G . G G G G    
637	. . G G G G G G G G G G G G . G G G G . G G . G . G G G . G G G G    
638	. . T T T T T T T T T T T T . T T T T . T T . T . T T T . T T T T    
639	. . G G G G G G G G G G G G . G G G G . G G . G . G G G . G G G G    
640	. . G G G G G G G G G G G G . G G G G . G G . G . G G G . G G G G    
641	. . C C C C C C C C C C C C . C C C C . C C . C . C C C . C C C C    
642	. . A A A A A A A A A A A A . A A A A . A A . A . A A A . A A A A    
643	. . G G G G G G G G G G G G . G G G G . G G . G . G G G . G G G G    
644	. . A A A A A A A A A A A A . A A A A A A A . A . A A A . A A A A    
645	. . C C C C C C C C C C C C . C C C C C C C . C . C C C . C C C C    
646	. . G G G G G G G G G G G G . G G G G G G G . G . G G G . G G G G    
647	. . C C C C C C C C C C C C . C C C C C C C . C . C C C . C C C C    
648	. . T T T T T T T T T T T T . T T T T T T T . T . T T T . T T T T    
649	. . A A A A A A A A A A A A . A A A A A A A . A . A A A A A A A A    
650	. . G G G G G G G G G G G G . G G G G G G G . G . G G G G G G G G    
651	. . C C C C C C C C C C C C . C C C C C C C . C . C C C C C C C C    
652	. . T T T T T T T T T T T T . T T T T T T T . T . T T T T T T T T    
653	. . C C C C C C C C C C C C . C C C C C C C . C . C C C C C C C C    
654	. . C C C C C C C C C C C C . C C C C C C C . C . C C C C C C C C    
655	. . A A A A A A A A A A A A . A A A A A A A . A . A A A A A A A A    
656	. . T T T T T T T T T T T T . T T T T T T T . T . T T T T T T T T    
657	. . A A A A A A A A A A A A . A A A A A A A . A . A A A A A A A A    
658	. . G G G G G G G G G G G G . G G G G G G G . G . G G G G G G G G    
659	. . A A A A A A A A A A A A . A A A A A A A . A . A A A A A A A A    
660	. . A A A A A A A A A A A A . A A A A A A A . A . A A A A A A A A    
661	. . T T T T T T T T T T T T . T T T T T T T . T . T T T T T T T T    
662	. . A A A A A A A A A A A A . A A A A A A A . A . A A A A A A A A    
663	. . C C C C C C C C C C C C . C C C C C C C . C . C C C C C C C C    
664	. . C C C C C C C C C C C C . C C C C C C C . C . C C C C C C C C    
665	. . T T T T T T T T T T T T . T T T T T T T . T . T T T T T T T T    
666	. . T T T T T T T T T T T T . T T T T T T T . T . T T T T T T T T    
667	. . T T T T T T T T T T T T . T T T T T T T . T . T T T T T T T T    
668	. . A A A A A A A A A A A A . A A A A A A A . A . A A A A A A A A    
669	. . A A A A A A A A A A A A . A A A A A A A . A . A A A A A A A A    
670	. . T T T T T T T T T T T T . T T T T T T T . T . T T T T T T T T    
671	. . G G G G G G G G G G G G . G G G G G G G . G . G G G G G G G G    
672	. . T T T T T T T T T T T T . T T T T T T T . T . T T T T T T T T    
673	. . A A A A A A A A A A A A . A A A A A A A . A . A A A A A A A A    
674	. . G G G G G G G G G G G G . G G G G G G G . G . G G G G G G G G    
675	. . G G G G G G G G G G G G . G G G G G G G . G . G G G G G G G G    
676	. . T T T T T T T T T T T T . T T T T T T T T T . T T T T T T T T    
677	. . . G G G G G G G G G G G . G G G G G G G G G . G G G G G G G G    
678	. . . T T T T T T T T T T T . T T T T T T T T T . T T T T T T T T    
679	. . . C C C C C C C C C C C . C C C C C C C C C . C C C C C C C C    
680	. . . C C C C C C C C C C C . C C C C C C C C C . C C C C C C C C    
681	. . . A A A A A A A A A A A . A A A A A A A A A . A A A A A A A A    
682	. . . T T T T T T T T T T T . T T T T T T T T T . T T T T T T T T    
683	. . . C C C C C C C C C C C . C C C C C C C C C . C C C C C C C C    
684	. . . A A A A A A A A A A A . A A A A A A A A A . A A A A A A A A    
685	. . . A A A A A A A A A A A . A A A A A A A A A . A A A A A A A A    
686	. . . A A A A A A A A A A A . A A A A A A A A A . A A A A A A A A    
687	. . . T T T T T T T T T T T . T T T T T T T T T . T T T T T T T T    
688	. . . T T T T T T T T T T T . T T T T T T T T T . T T T T T T T T    
689	. . . A A A A A A A A A A A . A A A A A A A A A . A A A A A A A A    
690	. . . G G G G G G G G G G G . G G G G G G G G G . G G G G G G G G    
691	. . . A A A A A A A A A A A . A A A A A A A A A . A A A A A A A A    
692	. . . A A A A A A A A A G A . A A A A A A A A A . A A A A A A A A    
693	. . . T T T T T T T T T T T . T T T T T T T T T . T T T T T T T T    
694	. . . A A A A A A A A A A A . A A A A A A A A A . A A A A A A A A    
695	. . . A A A A A A A A A A A . A A A A A A A A A . A A A A A A A A    
696	. . . G G G G G G G G G G G . G G G G G G G G G . G G G G G G G G    
697	. . . T T T T T T T T T T T . T T T T T T T T T . T T T T T T T T    
698	. . . T T T T T T T T T T T . T T T T T T T T T . T T T T T T T T    
699	. . . G G G G G G G G G G G . G G G G G G G G G . G G G G G G G G    
700	. . . T T T T T T T T T T T . T T T T T T T T T . T T T T T T T T    
701	. . . A A A A A A A A A A A . A A A A A A A A A . A A A A A A A A    
702	. . . A A A A A A A A A A A . A A A A A A A A A . A A A A A A A A    
703	. . . T T T T T T T T T T T . T T T T T T T T T . T T T T T T T T    
704	. . . T T T T T T T T T T T . T T T T T T T T T . T T T T T T T T    
705	. . . T T T T T T T T T T T . T T T T T T T T T . T T T T T T T T    
706	. . . G G G G G G G G G G G . G G G G G G G G G . G G G G G G G G    
707	. . . G G G G G G G G G G G . G G G G G G G G G . G G G G G G G G    
708	. . . G G G G G G G G G G G . G G G G G G G G G . G G G G G G G G    
709	. . . T T T T T T T T T T T . T T T T T T T T T . T T T T T T T T    
710	. . . G G G G G G G G G G G . G G G G G G G G G . G G G G G G G G    
711	. . . A A A A A A A A A A A . A A A A A A A A A . A A A A A A A A    
712	. . . A A A A A A A A A A A . A A A A A A A A A . A A A A A A A A    
713	. . . C C C C C C C C C C C . C C C C C C C C C . C C C C C C C C    
714	. . . C C C C C C C C C C C . C C C C C C C C C . C C C C C C C C    
715	. . . A A A A A A A A A A A . A A A A A A A A A . A A A A A A A A    
716	. . . T T T T T T T T T T T . T T T T T T T T T . T T T T T T T T    
717	. . . T T T T T T T T T T T . T T T T T T T T T . T T T T T T T T    
718	. . . T T T T T T T T T T T . T T T T T T T T T . T T T T T T T T    
719	. . . C C C C C C C C C C C . C C C C C C C C C . C C C C C C C C    
720	. . . T T T T T T T T T T T . T T T T T T T T T . T T T T T T T T    
721	. . . T T T T T T T T T T T . T T T T T T T T T . T T T T T T T T    
722	. . . T T T T T T T T T T T . T T T T T T T T T . T T T T T T T T    
723	. . . A A A A A A A A A A A . A A A A A A A A A . A A A A A A A A    
724	. . . A A A A A A A A A A A . A A A A A A A A A . A A A A A A A A    
725	. . . A A A A A A A A A A A . A A A A A A A A A . A A A A A A A A    
726	. . . G G G G G G G G G G G . G G G G G G G G G . G G G G G G G G    
727	. . . C C C C C C C C C C C . C C C C C C C C C . C C C C C C C C    
728	. . . A A A A A A A A A A A . A A A A A A A A A . A A A A A A A A    
729	. . . A A A A A A A A A A A . A A A A A A A A A . A A A A A A A A    
730	. . . A A A A A A A A A A A . A A A A A A A A A . A A A A A A A A    
731	. . . G G G G G G G G G G G . G G G G G G G G G . G G G G G G G G    
732	. . . G G G G G G G G G G G . G G G G G G G G G . G G G G G G G G    
733	. . . A A A A A A A A A A A . A A A A A A A A A . A A A A A A A A    
734	. . . A A A A A A A A A A A . A A A A A A A A A . A A A A A A A A    
735	. . . A A A A A A A A A A A . A A A A A A A A A . A A A A A A A A    
736	. . . A A A A A A A A A A A . A A A A A A A A A . A A A A A A A A    
737	. . . A A A A A A A A A A A . A A A A A A A A A . C C A A A A A A    
738	. . . C C C C C C C C C C C . C C C C C C C C C . C C C C C C C C    
739	. . . A A A A A A A A A A A . A A A A A A A A A . A A A A A A A A    
740	. . . T T T T T T T T T T T . T T T T T T T T T . T T T T T T T T    
741	. . . G G G G G G G G G G G . G G G G G G G G G . G G G G G G G G    
742	. . . A A A A A A A A A A A . A A A A A A A A A . A A A A A A A A    
743	. . . C C C C C C C C C C C . C C C C C C C C C . C C C C C C C C    
744	. . . G G G G G G G G G G G . G G G G G G G G G . A A G G G G G G    
745	. . . C C C C C C C C C C C . C C C C C C C C C . C C C C C C C C    
746	. . . T T T T T T T T T T T . T T T T T T T T T . T T T T T T T T    
747	. . . C C C C C C C C C C C . C C C C C C C C C . C C C C C C C C    
748	. . . T T T T T T T T T T T . T T T T T T T T T . T T T T T T T T    
749	. . . T T T T T T T T T T T . T T T T T T T T T . T T T T T T T T    
750	. . . G G G G G G G G G G G . G G G G G G G G G . G G G G G G G G    
751	. . . T T T T T T T T T T T . T T T T T T T T T . T T T T T T T T    
752	. . . T T T T T T T T T T T . T T T T T T T T T . T T T T T T T T    
753	. . . G G G G G G G G G G G . G G G G G G G G G . G G G G G G G G    
754	. . . A A A A A A A A A A A . A A A A A A A A A . A A A A A A A A    
755	. . . A A A A A A A A A A A . A A A A A A A A A . A A A A A A A A    
756	. . . T T T T T T T T T T T . T T T T T T T T T . T T T T T T T T    
757	. . . T T T T T T T T T T T . T T T T T T T A T . T T T T T T T T    
758	. . . A A A A A A A A A A A . A A A A A A A A A . A A A A A A A A    
759	. . . A A A A A A A A A A A . A A A A A A A A A . A A A A A A A A    
760	. . . A A A A A A A A A A A . A A A A A A A A A . A A A A A A A A    
761	. . . T T T T T T T T T T T . T T T T T T T T T . T T T T T T T T    
762	. . . T T T T T T T T T T T . T T T T T T T T T . T T T T T T T T    
763	. . . G G G G G G G G G G G . G G G G G G G G G . G G G G G G G G    
764	. . . T T T T T T T T T T T . T T T T T T T T T . T T T T T T T T    
765	. . . G G G G G G G G G G G . G G G G G G G G G . G G G G G G G G    
766	. . . T T T T T T T T T T T . T T T T T T T T T . T T T T T T T T    
767	. . . G G G G G G G G G G G . G G G G G G G G G . G G G G G G G G    
768	. . . T T T T T T T T T T T . T T T T T T T T T . T T T T T T T T    
769	. . . - - - - - - - - - - - . - - T T T T - - - . - - - - T T T T  8	 indel  
770	. . . - - - - - - - - - - - . - - T T T T - - - . - - - - T T T T  8	 indel  
771	. . . T T T T T T T T T T T . T T T T T T T T T T T T T T T T T T    
772	. . . A A A A A A A A A A A . A A A A A A A A A A A A A A A A A A    
773	. . . T T T T T T T T T T T . T T T T T T T T T T T T T T T T T T    
774	. . . A A A A A A A A A A A . A A A A A A A A A A A A A A A A A A    
775	. . . G G G G G G G G G G G . G G G G G G G G G G G G G G G G G G    
776	. . . T T T T T T T T T T T . T T T T T T T T T T T T T T T T T T    
777	. . . T T T T T T T T T T T . T T T T T T T T T T T T T T T T T T    
778	. . . A A A A A A A A A A A . A A A A A A A A A A A A A A A A A A    
779	. . . G G G G G G G G G G G . G G G G G G G G G G G G G G G G G G    
780	. . . T T T T T T T T T T T . T T T T T T T T T T T T T T T T T T    
781	. . . G G G G G G G G G G G . G G G G G G G G G G G G G G G G G G    
782	. . . G G G G G G G G G G G . G G G G G G G G G G G G G G G G G G    
783	. . . T T T T T T T T T T T . T T T T T T T T T T T T T T T T T T    
784	. . . A A A A A A A A A A A . A A A A A A A A A A A A A A A A A A    
785	. . . T T T T T T T T T T T . T T T T T T T T T T T T T T T T T T    
786	. . . G G G G G G G G G G G . G G G G G G G G G G G G G G G G G G    
787	. . . C T T T T T T T T T T . T T T T T T T T T T T T T T T T T T    
788	. . . . G G G G G G G G G G . G G G G G G G G G G G G G G G G G G    
789	. . . . T T T T T T T T T T . T T T T T T T T T T T T T T T T T T    
790	. . . . T T T T T T T T T T . T T T T T T T T T T T T T T T T T T    
791	. . . . G G G G G G G G G G . G G G G G G G G G G G G G G G G G G    
792	. . . . A A A A A A A A A A . A A A A A A A A A A A A A A A A A A    
793	. . . . T T T T T T T T T T . T T T T T T T T T T T T T T T T T T    
794	. . . . T T T T T T T T T T . T T T T T T T T T T T T T T T T T T    
795	. . . . A A A A A A A A A A . A A A A A A A A A A A A A A A A A A    
796	. . . . T T T T T T T T T T . T T T T T T T T T T T T T T T T T T    
797	. . . . G G G G G G G G G G . G G G G G G G G G G G G G G G G G G    
798	. . . . A A A A A A A A A A . A A A A A A A A A A A A A A A A A A    
799	. . . . A A A A A A A A A A . A A A A A A A A A A A A A A A A A A    
800	. . . . G G G G G G G G G G . G G G G G G G G G G G G G G G A A G    
801	. . . . C C C C C C C C C C . C C C C C C C C C C C C C C C C C C    
802	. . . . A A A A A A A A A A . A A A A A A A A A A A A A A A A A A    
803	. . . . T T T T T T T T T T . T T T T T T T T T T T T T T T T T T    
804	. . . . T T T T T T T T T T . T T T T T T T T T T T T T T T T T T    
805	. . . . G G G G G G G G G G . G G G G G G G G G G G G G G G G G G    
806	. . . . C C C C C C C C C C . C C C C C C C C C C C C C C C C C C    
807	. . . . A A A A A A A A A A . A A A A A A A A A A A A A A A A A A    
808	. . . . C C C C C C C C C C . C C C C C C C C C C C C C C C C C C    
809	. . . . T T T T T T T T T T . T T T T T T T T T T T T T T T T T T    
810	. . . . T T T T T T T T T T . T T T T T T T T T T T T T T T T T T    
811	. . . . G G G G G G G G G G . G G G G G G G G G G G G G G G G G G    
812	. . . . T T T T T T T T T T . T T T T T T T T T T T T T T T T T T    
813	. . . . C C C C C C C C C C . C C C C C C C C C C C C C C C C C C    
814	. . . . A A A A A A A A A A . A A A A A A A A A A A A A A A A A A    
815	. . . . T T T T T T T T T T . T T T T T T T T T T T T T T T T T T    
816	. . . . C C C C C C C C C C . C C C C C C C C C C C C C C C C C C    
817	. . . . T T T T T T T T T T . T T T T T T T T T T T T T T T T T T    
818	. . . . A A A A A A A A A A . A A A A A A A A A A A A A A A A A A    
819	. . . . C C C C C C C C C C . C C C C C C C C C C C C C C C C C C    
820	. . . . A A A A A A A A A A . A A A A A A A A A A A A A A A A A A    
821	. . . . A A A A A A A A A A . A A A A A A A A A A A A A A A A A A    
822	. . . . A A A A A A A A A A . A A A A A A A A A A A A A A A A A A    
823	. . . . T T T T T T T T T T . T T T T T T T T T T T T T T T T T T    
824	. . . . C C C C C C C C C C . C C C C C C C C C C C C C C C C C C    
825	. . . . C C C C C C C C C C . C C C C C C C C C C C C C C C C C C    
826	. . . . A A A A A A A A A A . A A A A A A A A A A A A A A A A A A    
827	. . . . T T T T T T T T T T . T T T T T T T T T T T T T T T T T T    
828	. . . . T T T T T T T T T T . T T T T T T T T T T T T T T T T T T    
829	. . . . T T T T T T T T T T . T T T T T T T T T T T T T T T T T T    
830	. . . . C C C C C C C C C C . C C C C C C C C C C C C C C C C C C    
831	. . . . T T T T T T T T T T T T T T T T T T T T T T T T T T T T T    
832	. . . . C C C C C C C C C C C C C C C C C C C C C C C C C C C C C    
833	. . . . A A A A A A A A A A A A A A A A A A A A A A A A A A A A A    
834	. . . . T T T T T T T T T T T T T T T T T T T T T T T T T T T T T    
835	. . . . T T T T T T T T T T T T T T T T T T T T T T T T T T T T T    
836	. . . . T T T T T T T T T T T T T T T T T T T T T T T T T T T T T    
837	. . . . T T T T T T T T T T T T T T T T T T T T T T T T T T T T T    
838	. . . . A A A A A A A A A A A A A A A A A A A A A A A A A A A A A    
839	. . . . C C C C C C C C C C C C C C C C C C C C C C C C C C C C C    
840	. . . . A A A A A A A A A A A A A A A A A A A A A A A A A A A A A    
841	. . . . A A A A A A A A A A A A A A A A A A A A A A A A A A A A A    
842	. . . . G G G G G G G G G G G G G G G G G G G G G G G G G G G G G    
843	. . . . T T T T T T T T T T T T T T T T T T T T T T T T T T T T T    
844	. . . . A A A A A A A A A A A A A A A A A A A A A A A A A A A A A    
845	. . . . A A A A A A A A A A A A A A A A A A A A A A A A A A A A A    
846	. . . . A A A A A A A A A A A A A A A A A A A A A A A A A A A A A    
847	. . . . C C C C C C C C C C C C C C C C C C C C C C C C C C C C C    
848	. . . . C C C C C C C C C C C C C C C C C C C C C C C C C C C C C    
849	. . . . T T T T T T T T T T T T T T T T T T T T T T T T T T T T T    
850	. . . . T T T T T T T T T T T T T T T T T T T T T T T T T T T T T    
851	. . . . T T T T T T T T T T T T T T T T T T T T T T T T T T T T T    
852	. . . . T T T T T T T T T T T T T T T T T T T T T T T T T T T T T    
853	. . . . T T T T T T T T T T T T T T T T T T T T T T T T T T T T T    
854	. . . . T T T T T T T T T T T T T T T T T T T T T T T T T T T T T    
855	. . . . A A A A A A A A A A A A A A A A A A A A A A A A A A A A A    
856	. . . . G G G G G G G G G G G G G G G G G G G G G G G G G G G G G    
857	. . . . T T T T T T T T T T T T T T T T T T T T T T T T T T T T T    
858	. . . . C C C C C C C C C C C C C C C C C C C C C C C C C C C C C    
859	. . . . T T T T T T T T T T T T T T T T T T T T T T T T T T T T T    
860	. . . . A A A A A A A A A A A A A A A A A A A A A A A A A A A A A    
861	. . . . G G G G G G G G G G G G G G G G G G G G G G G G G G G G G    
862	. . . . T T T T T T T T T T T T T T T T T T T T T T T T T T T T T    
863	. . . . C C C C C C C C C C C C C C C C C C C C C C C C C C C C C    
864	. . . . T T T T T T T T T T T T T T T T T T T T T T T T T T T T T    
865	. . . . T T T T T T T T T T T T T T T T T T T T T T T T T T T T T    
866	. . . . C C C C C C C C C C C C C C C C C C C C C C C C C C C C C    
867	. . . . A A A A A A A A A A A A A A A A A A A A A A A A A A A A A    
868	. . . . T T T T T T T T T T T T T T T T T T T T T T T T T T T T T    
869	. . . . T T T T T T T T T T T T T T T T T T T T T T T T T T T T T    
870	. . . . T T T T T T T T T T T T T T T T T T T T T T T T T T T T T    
871	. . . . A A A A A A A A A A A A A A A A A A A A A A A A A A A A A    
872	. . . . A A A A A A A A A A A A A A A A A A A A A A A A A A A A A    
873	. . . . T T T T T T T T T T T T T T T T T T T T T T T T T T T T T    
874	. . . . C C C C C C C C C C C C C C C C C C C C C C C C C C C C C    
875	. . . . A A A A A A A A A A A A A A A A A A A A A A A A A A A A A    
876	. . . . G G G G G G G G G G G G G G G G G G G G G G G G G G G G G    
877	. . . . T T T T T T T T T T T T T T T T T T T T T T T T T T T T T    
878	. . . . T T T T T T T T T T T T T T T T T T T T T T T T T T T T T    
879	. . . . T T T T T T T T T T T T T T T T T T T T T T T T T T T T T    
880	. . . . G G G G G G G G G G G G G G G G G G G G G G G G G G G G G    
881	. . . . G G G G G G G G G G G G G G G G G G G G G G G G G G G G G    
882	. . . . G G G G G G G G G G G G G G G G G G G G G G G G G G G G G    
883	. . . . T T T T T T T T T T T T T T T T T T T T T T T T T T T T T    
884	. . . . C C C C C C C C C C C C C C C C C C C C C C C C C C C C C    
885	. . . . T T T T T T T T T T T T T T T T T T T T T T T T T T T T T    
886	. . . . A A A A A A A A A A A A A A A A A A A A A A A A A A A A A    
887	. . . . A A A A A A A A A A A A A A A A A A A A A A A A A A A A A    
888	. . . . T T T T T T T T T T T T T T T T T T T T T T T T T T T T T    
889	. . . . T T T T T T T T T T T T T T T T T T T T T T T T T T T T T    
890	. . . . A A A A A A A A A A A A A A A A A A A A A A A A A A A A A    
891	. . . . A A A A A A A A A A A A A A A A A A A A A A A A A A A A A    
892	. . . . T T T T T T T T T T T T T T T T T T T T T T T T T T T T T    
893	. . . . T T T T T T T T T T T T T T T T T T T T T T T T T T T T T    
894	. . . . T T T T T T T T T T T T T T T T T T T T T T T T T T T T T    
895	. . . . A A A A A A A A A A A A A A A A A A A A A A A A A A A A A    
896	. . . . A A A A A A A A A A A A A A A A A A A A A A A A A A A A A    
897	. . . . A A A A A A A A A A A A A A A A A A A A A A A A A A A A A    
898	. . . . A A A A A A A A A A A A A A A A A A A A A A A A A A A A A    
899	. . . . T T T T T T T T T T T T T T T T T T T T T T T T T T T T T    
900	. . . . C C C C C C C C T C C C C C C C C C C C C C C T C C C C C    
901	. . . . C A A A A A A A A A A A A A A A A A A A A A A A A A A A A    
902	. . . . . A A A A A A A A A A A A A A A A A A A A A A A A A A A A    
903	. . . . . T T T T T T T T T T T T T T T T T T T T T T T T T T T T    
904	. . . . . T T T T T T T T T T T T T T T T T T T T T T T T T T T T    
905	. . . . . T T T T T T T T T T T T T T T T T T T T T T T T T T T T    
906	. . . . . C C C C C C C C C C C C C C C C C C C C C C C C C C C C    
907	. . . . . A A A A A A A A A A A A A A A A A A A A A A A A A A A A    
908	. . . . . T T T T T T T T T T T T T T T T T T T T T T T T T T T T    
909	. . . . . G G G G G G G G G G G G G G G G G G G G G G G G G G G G    
910	. . . . . C C C C C C C C C C C C C C C C C C C C C C C C C C C C    
911	. . . . . C C C C C C C C C C C C C C C C C C C C C C C C C C C C    
912	. . . . . A A A A A A A A A A A A A A A A A A A A A A A A A A A A    
913	. . . . . A A A A A A A A A A A A A A A A A A A A A A A A A A A A    
914	. . . . . A A A A A A A A A A A A A A A A A A A A A A A A A A A A    
915	. . . . . A A A A A A A A A A A A A A A A A A A A A A A A A A A A    
916	. . . . . T T T T T T T T T T T T T T T T T T T T T T T T T T T T    
917	. . . . . A A A A A A A A A A A A A A A A A A A A A A A A A A A A    
918	. . . . . T T T T T T T T T T T T T T T T T T T T T T T T T T T T    
919	. . . . . A A A A A A A A A A A A A A A A A A A A A A A A A A A A    
920	. . . . . A A A A A A A A A A A A A A A A A A A A A A A A A A A A    
921	. . . . . C C C C C C C C C C C C C C C C C C C C C C C C C C C C    
922	. . . . . A A A A A A A A A A A A A A A A A A A A A A A A A A A A    
923	. . . . . A A A A A A A A A A A A A A A A A A A A A A A A A A A A    
924	. . . . . T T T T T T T T T T T T T T T T T T T T T T T T T T T T    
925	. . . . . A A A A A A A A A A A A A A A A A A A A A A A A A A A A    
926	. . . . . T T T T T T T T T T T T T T T T T T T T T T T T T T T T    
927	. . . . . G G G G G G G G G G G G G G G G G G G G G G G G G G G G    
928	. . . . . A A A A A A A A A C A A A A A A A A A A A A A A A A A A    
929	. . . . . C C C C C C C C C C C C C C C C C C C C C C C C C C C C    
930	. . . . . A A A A A A A A A A A A A A A A A A A A A A A A A A A A    
931	. . . . . T T T T T T T T T T T T T T T T T T T T T T T T T T T T    
932	. . . . . T T T T T T T T T T T T T T T T T T T T T T T T T T T T    
933	. . . . . C C C C C C C C C C C C C C C C C C C C C C C C C C C C    
934	. . . . . A A A A A A A A A A A A A A A A A A A A A A A A A A A A    
935	. . . . . T T T T T T T T T T T T T T T T T T T T T T T G T T T T    
936	. . . . . T T T T T T T T T T T T T T T T T T T T T T T T T T T T    
937	. . . . . T T T T T T T T T T T T T T T T T T T T T T T T T T T T    
938	. . . . . A A A A A A A A A A A A A A A A A A A A A A A A A A A A    
939	. . . . . A A A A A A A A A A A A A A A A A A A A A A A A A A A A    
940	. . . . . T T T T T T T T T T T T T T T T T T T T T T T T T T T T    
941	. . . . . A A A A A A A A A A A A A A A A A A A A A A A A A A A A    
942	. . . . . C C C C C C C C C C C C C C C C C C C C C C C C C C C C    
943	. . . . . A A A A A A A A A A A A A A A A A A A A A A A A A A A A    
944	. . . . . A A A A A A A A A A A A A A A A A A A A A A A A A A A A    
945	. . . . . A A A A A A A A A A A A A A A A A A A A A A A A A A A A    
946	. . . . . A A A A A A A A A A A A A A A A A A A A A A A A A A A A    
947	. . . . . T T T T T T T T T T T T T T T T T T T T T T T T T T T T    
948	. . . . . G G G G G G G G G G G G G G G G G G G G G G G G G G G G    
949	. . . . . T T T T T T T T T T T T T T T T T T T T T T T T T T T T    
950	. . . . . A A A A A A A A A A A A A A A A A A A A A A A A A A A A    
951	. . . . . T T T T T T T T T T T T T T T T T T T T T T T T T T T T    
952	. . . . . T T T T T T T T T T T T T T T T T T T T T T T T T T T T    
953	. . . . . C C C C C C C C C C C C C C C C C C C C C C C C C C C C    
954	. . . . . A A A A A A A A A A A A A A A A A A A A A A A A A A A A    
955	. . . . . G G G G G G G G G G G G G G G G G G G G G G G G G G G G    
956	. . . . . T T T T T T T T T T T T T T T T T T T T T T T T T T T T    
957	. . . . . T T T T T T T T T T T T T T T T T T T T T T T T T T T T    
958	. . . . . T T T T T T T T T T T T T T T T T T T T T T T T T T T T    
959	. . . . . T T T T T T T T T T T T T T T T T T T T T T T T T T T T    
960	. . . . . A A A A A A A A A A A A A A A A A A A A A A A A A A A A    
961	. . . . . C C C C C C C C C C C C C C C C C C C C C C C C C C C C    
962	. . . . . A A A A A A A A A A A A A A A A A A A A A A A A A A A A    
963	. . . . . A A A A A A A A A A A A A A A A A A A A A A A A A A A A    
964	. . . . . C C C C C C C C C C C C C C C C C C C C C C C C C C C C    
965	. . . . . T T T T T T T T T T T T T T T T T T T T T T T T T T T T    
966	. . . . . T T T T T T T T T T T T T T T T T T T T T T T T T T T T    
967	. . . . . C C C C C C C C C C C C C C C C C C C C C C C C C C C C    
968	. . . . . A A A A A A A A A A A A A A A A A A A A A A A A A A A A    
969	. . . . . C C C C C C C C C C C C C C C C C C C C C C C C C C C C    
970	. . . . . T T T T T T T T T T T T T T T T T T T T T T T T T T T T    
971	. . . . . T T T T T T T T T T T T T T T T T T T T T T T T T T T T    
972	. . . . . C C C C C C C C C T C C C C C C C C C C C C C C T C C C    
973	. . . . . T T T T T T T T T T T T T T T T T T T T T T T T T T T T    
974	. . . . . G G G G G G G G G G G G G G G G G G G G G G G G G G G G    
975	. . . . . A A A A A A A A A A A A A A A A A A A A A A A A A A A A    
976	. . . . . T T T T T T T T T T T T T T T T T T T T T T T T T T T T    
977	. . . . . T T T T T T T T T T T T T T T T T T T T T T T T T T T T    
978	. . . . . T T T T T T T T T T T T T T T T T T T T T T T T T T T T    
979	. . . . . T T T T T T T T T T T T T T T T T T T T T T T T T T T T    
980	. . . . . T T T T T T T T T T T T T T T T T T T T T T T T T T T T    
981	. . . . . G G G G G G G G G G G G G G G G G G G G G G G G G G G G    
982	. . . . . C C C C C C C C C C C C C C C C C C C C C C C C C C C C    
983	. . . . . T T T T T T T T T T T T T T T T T T T T T T T T T T T T    
984	. . . . . G G G G G G G G G G G G G G G G G G G G G G G G G G G G    
985	. . . . . G G G G G G G G G G G G G G G G G G G G G G G G G G G G    
986	. . . . . A A A A A A A A A A A A A A A A A A A A A A A A A A A A    
987	. . . . . A A A A A A A A A A A A A A A A A A A A A A A A A A A A    
988	. . . . . T T T T T T T T T T T T T T T T T T T T T T T T T T T T    
989	. . . . . T T T T T T T T T T T T T T T T T T T T T T T T T T T T    
990	. . . . . T T T T T T T T T T T T T T T T T T T T T T T T T T T T    
991	. . . . . A A A A A A A A A A A A A A A A A A A A A A A A A A A A    
992	. . . . . A A A A A A A A A A A A A A A A A A A A A A A A A A A A    
993	. . . . . A A A A A A A A A A A A A A A A A A A A A A A A A A A A    
994	. . . . . T T T T T T T T T T T T T T T T T T T T T T T T T T T T    
995	. . . . . A A A A A A A A A A A A A A A A A A A A A A A A A A A A    
996	. . . . . C C C C C C C C C C C C C C C C C C C C C C C C C C C C    
997	. . . . . A A A A A A A A A A A A A A A A A A A A A A A A A A A A    
998	. . . . . T T T T T T T T T T T T T T T T T T T T T T T T T T T T    
999	. . . . . A A A A A A A A A A A A A A A A A A A A A A A A A A A A    
1000	. . . . . T T T T T T T T T T T T T T T T T T T T T T T T T T T T    
1001	. . . . . A A A A A A A A A A A A A A A A A A A A A A A A A A A A    
1002	. . . . . T T T T T T T T T T T T T T T T T T T T T T T T T T T T    
1003	. . . . . G G G G G G G G G G G G G G G G G G G G G G G G G G G G    
1004	. . . . . T T T T T T T T T T T T T T T T T T T T T T T T T T T T    
1005	. . . . . T T T T T T T T T T T T T T T T T T T T T T T T T T T T    
1006	. . . . . C C C C C C C C C T C C C C C C C C C C C C C C C C C C    
1007	. . . . . T T T T T T T T T T T T T T T T T T T T T T T T T T T T    
1008	. . . . . A A A A A A A A A A A A A A A A A A A A A A A A A A A A    
1009	. . . . . T T T T T T T T T T T T T T T T T T T T T T T T T T T T    
1010	. . . . . G G G G G G G G G G G G G G G G G G G G G G G G G G G G    
1011	. . . . . T T T T T T T T T T T T T T T T T T T T T T T T T T T T    
1012	. . . . . G G G G G G G G G G G G G G G G G G G G G G G G G G G G    
1013	. . . . . A A A A A A A A A A A A A A A A A A A A A A A A A A A A    
1014	. . . . . A A A A A A A A A A A A A A A A A A A A A A A A A A A A    
1015	. . . . . A A A A A A A A A A A A A A A A A A A A A A A A A A A A    
1016	. . . . . T T T T T T T T T T T T T T T T T T T T T T T T T T T T    
1017	. . . . . A A A A A A A A A A A A A A A A A A A A A A A A A A A A    
1018	. . . . . A A A A A A A A A A A A A A A A A A A A A A A A A A A A    
1019	. . . . . G G G G G G G G G G G G G G G G G G G G G G G G G G G G    
1020	. . . . . T T T T T T T T T T T T T T T T T T T T T T T T T T T T    
1021	. . . . . A A A A A A A A A A A A A A A A A A A A A A A A A A A A    
1022	. . . . . T T T T T T T T T T T T T T T T T T T T T T T T T T T T    
1023	. . . . . T T T T T T T T T T T T T T T T T T T T T T T T T T T T    
1024	. . . . . T T T T T T T T T T T T T T T T T T T T T T T T T T T T    
1025	. . . . . T T T T T T T T T T T T T T T T T T T T T T T T T T T T    
1026	. . . . . A A A A A A A A A A A A A A A A A A A A A A A A A A A A    
1027	. . . . . T T T T T T T T T T T T T T T T T T T T T T T T T T T T    
1028	. . . . . A A A A A A A A A A A A A A A A A A A A A A A A A A A A    
1029	. . . . . A A A A A A A A A A A A A A A A A A A A A A A A A A A A    
1030	. . . . . A A A A A A A A A A A A A A A A A A A A A A A A A A A A    
1031	. . . . . T T T T T T T T T T T T T T T T T T T T T T T T T T T T    
1032	. . . . . A A A A A A A A A A A A A A A A A A A A A A A A A A A A    
1033	. . . . . T T T T T T T T T T T T T T T T T T T T T T T T T T T T    
1034	. . . . . A A A A A A A A A A A A A A A A A A A A A A A A A A A A    
1035	. . . . . T T T T T T T T T T T T T T T T T T T T T T T T T T T T    
1036	. . . . . G G G G G G G G G G G G G G G G G G G G G G G G G G G G    
1037	. . . . . C C C C C C C C C C C C C C C C C C C C C C C C C C C C    
1038	. . . . . T T T T T T T T T T T T T T T T T T T T T T T T T T T T    
1039	. . . . . A A A A A A A A A A A A A A A A A A A A A A A A A A A A    
1040	. . . . . C C C C C C C C C C C C C C C C C C C C C C C C C C C C    
1041	. . . . . T T T T T T T T T T T T T T T T T T T T T T T T T T T T    
1042	. . . . . A A A A A A A A A A A A A A A A A A A A A A A A A A A A    
1043	. . . . . T T T T T T T T T T T T T T T T T T T T T T T T T T T T    
1044	. . . . . A A A A A A A A A A A A A A A A A A A A A A A A A A A A    
1045	. . . . . T T T T T T T T T T T T T T T T T T T T T T T T T T T T    
1046	. . . . . A T T T T T T T T T T T T T T T T T T T T T T T T T T T    
1047	. . . . . . A A A . A A A A A A A A A A A A A A A A A A A A A A A    
1048	. . . . . . A A A . A A A A A A A A A A A A A A A A A A A A A A A    
1049	. . . . . . A A A . A A A A A A A A A A A A A A A A A A A A A A A    
1050	. . . . . . T A T . T T T T T T T T T T T T T T T T T T T T T T T    
1051	. . . . . . A . A . A A A A A A A A A A A A A A A A A A A A A A A    
1052	. . . . . . A . A . A A A A A A A A A A A A A A A A A A A A A A A    
1053	. . . . . . A . A . A A A A A A A A A A A A A A A A A A A A A A A    
1054	. . . . . . A . A . A A A A A A A A A A A A A A A A A A A A A A A    
1055	. . . . . . T . T . T T T T T T T T T T T T T T T T T T T T T T T    
1056	. . . . . . A . A . A A A A A A A A A A A A A A A A A A A A A A A    
1057	. . . . . . A . A . A A A A A A A A A A A A A A A A A A A A A A A    
1058	. . . . . . A . A . A A A A A A A A A A A A A A A A A A A A A A A    
1059	. . . . . . A . A . A A A A A A A A A A A A A A A A A A A A A A A    
1060	. . . . . . C . C . C C C C C C C C C C C C C C C C C C C C C C C    
1061	. . . . . . C . C . C C C C C C C C C C C C C C C C C C C C C C C    
1062	. . . . . . A . A . A A A A A A A A A A A A A A A A A A A A A A A    
1063	. . . . . . T . T . T T T T T T T T T T T T T T T T T T T T T T T    
1064	. . . . . . C . C . C C C C C C C C C C C C C C C C C C C C C C C    
1065	. . . . . . A . A . A A A A A A A A A A A A A A A A A A A A A A A    
1066	. . . . . . G . G . G G G G G G G G G G G G G G G G G G G G G G G    
1067	. . . . . . T . T . T T T T T T T T T T T T T T T T T T T T T T T    
1068	. . . . . . G . G . G G G G G G G G G G G G G G G G G G G G G G G    
1069	. . . . . . C . C . C C C C C C C C C C C C C C C C C C C C C C C    
1070	. . . . . . C . C . C C C C C C C C C C C C C C C C C C C C C C C    
1071	. . . . . . . . T . T T T T T T T T T T T T T T T T T T T T T T T    
1072	. . . . . . . . . . . G G G G G G G G G G G G G G G G G G G G G G    
1073	. . . . . . . . . . . A A A A A A A A A A A A A A A A A A A A A A    
1074	. . . . . . . . . . . A A A A A A A A A A A A A A A A A A A A A A    
1075	. . . . . . . . . . . A A A A A A A A A A A A A A A A A A A A A A    
1076	. . . . . . . . . . . T T T T T T T T T T T T T T T T T T T T T T    
1077	. . . . . . . . . . . G G G G G G G G G G G G G G G G G G G G G G    
1078	. . . . . . . . . . . G G G G G G G G G G G G G A G G G G G G G G    
1079	. . . . . . . . . . . C C C C C C C C C C C C C C C C C C C C C C    
1080	. . . . . . . . . . . T T T T T T T T T T T T C T T T T T T T T T    
1081	. . . . . . . . . . . T T T T T T T T T T T T T T T T T T T T T T    
1082	. . . . . . . . . . . C C C C C C C C C C C C C C C C C C C C C C    
1083	. . . . . . . . . . . A C A A A A A A A A A A A A A A A A A A A A    
1084	. . . . . . . . . . . A . G G G G G G G G G G G G G G G G G G G G    
1085	. . . . . . . . . . . . . A A A A A A A A A A A A A A A A A A A A    
1086	. . . . . . . . . . . . . A A A A A A A A A A A A A A A A A A A A    
1087	. . . . . . . . . . . . . C C C C C C C C C C C C C C C C C C C C    
1088	. . . . . . . . . . . . . C C C C C C C C C C C C C C C C C C C C    
1089	. . . . . . . . . . . . . T T T T T T T T T T T T T T T T T T T T    
1090	. . . . . . . . . . . . . T T T T T T T T T T T T T T T T T T T T    
1091	. . . . . . . . . . . . . T T T T T T T T T T T T T T T T T T T T    
1092	. . . . . . . . . . . . . C T C C C C C C C C C C C C C C C C C C    
1093	. . . . . . . . . . . . . T . T T T T T T T T T T T T T T T T T T    
1094	. . . . . . . . . . . . . G . G G G G G G G G G G G G G G G G G G    
1095	. . . . . . . . . . . . . A . A A A A A A A A A A A A A A A A A A    
1096	. . . . . . . . . . . . . T . T T T T T T T T T T T T T T T T T T    
1097	. . . . . . . . . . . . . C . C C C C C C C C C C C C C C C C C C    
1098	. . . . . . . . . . . . . A . A A A A A C A A A A A A A A A A A A    
1099	. . . . . . . . . . . . . C . C C C C C C C C C C C C C C C C C C    
1100	. . . . . . . . . . . . . C . C C C C C C C C C C C C C C C C C C    
1101	. . . . . . . . . . . . . A . A A A A A C A A A A A A A A C A A A    
1102	. . . . . . . . . . . . . C . C C C C C C C C C C C C C C C C C C    
1103	. . . . . . . . . . . . . C . C C C C C C C C C C C C C C C C C C    
1104	. . . . . . . . . . . . . T . T T T T T T T T T T T T T T T T T T    
1105	. . . . . . . . . . . . . G . G G G G G G G G G G G G G G G G G G    
1106	. . . . . . . . . . . . . T . T T T T T T T T T T T T T T T T T T    
1107	. . . . . . . . . . . . . A . A A A A A A A A A A A A A A A A A A    
1108	. . . . . . . . . . . . . A . A A A A A A A A A A A A A A A A A A    
1109	. . . . . . . . . . . . . A . A A A A A A A A A A A A A A A A A A    
1110	. . . . . . . . . . . . . A . A A A A A A A A A A A A A A A A A A    
1111	. . . . . . . . . . . . . C . C C C C C C C C C C C C C C C C C C    
1112	. . . . . . . . . . . . . A . A A A A A A A A A A A A A A A A A A    
1113	. . . . . . . . . . . . . A . A A A A A A A A A A A A A A A A A A    
1114	. . . . . . . . . . . . . A . A A A A A A A A A A A A A A A A A A    
1115	. . . . . . . . . . . . . C . C C C C C C C C C C C C C C C C C C    
1116	. . . . . . . . . . . . . T . T T T T T T T T T T T T T T T T T T    
1117	. . . . . . . . . . . . . G . G G G G G G G G G G G G G G G G G G    
1118	. . . . . . . . . . . . . A . A A A A A A A A A A A A A A A A A A    
1119	. . . . . . . . . . . . . T . T T T T T T T T T T T T T T T T T T    
1120	. . . . . . . . . . . . . T . T T T T T T T T T T T T T T T T T T    
1121	. . . . . . . . . . . . . G . G G G G G G G G G G G G G G G G G G    
1122	. . . . . . . . . . . . . A . A A A A A A A A A A A A A A A A A A    
1123	. . . . . . . . . . . . . G . G G G G G G G G G G G G G G G G G G    
1124	. . . . . . . . . . . . . G . G G G G G G G G G G G G G G G G G G    
1125	. . . . . . . . . . . . . T . T T T T T T T T T T T T T T T T T T    
1126	. . . . . . . . . . . . . C . T T T T T T T T T T T T T T T T T T    
1127	. . . . . . . . . . . . . . . T T T T T T T T T T T T T T T T T T    
1128	. . . . . . . . . . . . . . . T T T T T T T T T T T T T T T T T T    
1129	. . . . . . . . . . . . . . . G G G G G G G G G G G G G G G G G G    
1130	. . . . . . . . . . . . . . . A A A A A A A A A A A A A A A A A A    
1131	. . . . . . . . . . . . . . . T T T T T T T T T T T T T T T T T T    
1132	. . . . . . . . . . . . . . . G G G G G G G G G G G G G G G G G G    
1133	. . . . . . . . . . . . . . . C C C C C C C C C C C C C C C C C C    
1134	. . . . . . . . . . . . . . . T T T T T T T T T T T T T T T T T T    
1135	. . . . . . . . . . . . . . . G G G G G G G G G G G G G G G G G G    
1136	. . . . . . . . . . . . . . . A A A A A A A A A A A A A A A A A A    
1137	. . . . . . . . . . . . . . . C C C C C C C C C C C C C C C C C C    
1138	. . . . . . . . . . . . . . . A A A A A A A A A A A A A A A A A A    
1139	. . . . . . . . . . . . . . . C C C C C C C C C C C C C C C C C C    
1140	. . . . . . . . . . . . . . . G G G G G G G G G G G G G G G G G G    
1141	. . . . . . . . . . . . . . . A A A A A A A A A A A A A A A A A A    
1142	. . . . . . . . . . . . . . . T T T T T T T T T T T T T T T T T T    
1143	. . . . . . . . . . . . . . . A A A A A A A A A A A A A A A A A A    
1144	. . . . . . . . . . . . . . . C C C C C C C C C C C C C C C C C C    
1145	. . . . . . . . . . . . . . . A A A A A A A A A A A A A A A A A A    
1146	. . . . . . . . . . . . . . . T T T T T T T T T T T T T T T T T T    
1147	. . . . . . . . . . . . . . . A A A A A A A A A A A A A A A A A A    
1148	. . . . . . . . . . . . . . . T T T T T T T T T T T T T T T T T T    
1149	. . . . . . . . . . . . . . . T T T T T T T T T T T T T T T T T T    
1150	. . . . . . . . . . . . . . . T T T T T T T T T T T T T T T T T T    
1151	. . . . . . . . . . . . . . . T T T T T T T T T T T T T T T T T T    
1152	. . . . . . . . . . . . . . . T T T T T T T T T T T T T T T T T T    
1153	. . . . . . . . . . . . . . . G G G G G G G G G G G G G G G G G G    
1154	. . . . . . . . . . . . . . . T T T T T T T T T T T T T T T T T T    
1155	. . . . . . . . . . . . . . . A . . . . A A A A A A A A A A A A A    
1156	. . . . . . . . . . . . . . . A . . . . A A A A A A A A A A A A A    
1157	. . . . . . . . . . . . . . . A . . . . A A A A A A A A A A A A A    
1158	. . . . . . . . . . . . . . . C . . . . C C C C C C C C C C C C C    
1159	. . . . . . . . . . . . . . . A . . . . A A A A A A A A A A A A A    
1160	. . . . . . . . . . . . . . . A . . . . A A A A A A A A A A A A A    
1161	. . . . . . . . . . . . . . . A . . . . A A A A A A A A A A A A A    
1162	. . . . . . . . . . . . . . . A . . . . A A A A A A A A A A A A A    
1163	. . . . . . . . . . . . . . . T . . . . T T T T T T T T T T T T T    
1164	. . . . . . . . . . . . . . . G . . . . G G G G G G G G G G G G G    
1165	. . . . . . . . . . . . . . . A . . . . A A A A A A A A A A A A A    
1166	. . . . . . . . . . . . . . . T . . . . T T T T T T T T T T T T T    
1167	. . . . . . . . . . . . . . . G . . . . G G G G G G G G G G G G G    
1168	. . . . . . . . . . . . . . . G . . . . G G G G G G G G G G G G G    
1169	. . . . . . . . . . . . . . . A . . . . A A A A A A A A A A A A A    
1170	. . . . . . . . . . . . . . . T . . . . T T T T T T T T T T T T T    
1171	. . . . . . . . . . . . . . . G . . . . G G G G G G G G G G G G G    
1172	. . . . . . . . . . . . . . . A . . . . A A A A A A A A A A A A A    
1173	. . . . . . . . . . . . . . . T . . . . T T T T T T T T T T T T T    
1174	. . . . . . . . . . . . . . . T . . . . T T T T T T T T T T T T T    
1175	. . . . . . . . . . . . . . . T . . . . T T T T T T T T T T T T T    
1176	. . . . . . . . . . . . . . . G . . . . G G G G G G G G G G G G G    
1177	. . . . . . . . . . . . . . . T . . . . T T T T T T T T T T T T T    
1178	. . . . . . . . . . . . . . . A . . . . A A A A A A A A A A A A A    
1179	. . . . . . . . . . . . . . . T . . . . T T T T T T T T T T T T T    
1180	. . . . . . . . . . . . . . . A . . . . A A A A A A A A A A A A A    
1181	. . . . . . . . . . . . . . . A . . . . A A A A A A A A A A A A A    
1182	. . . . . . . . . . . . . . . A . . . . A A A A A A A A A A A A A    
1183	. . . . . . . . . . . . . . . T . . . . T T T T T T T T T T T T T    
1184	. . . . . . . . . . . . . . . A . . . . A A A A A A A A A A A A A    
1185	. . . . . . . . . . . . . . . A . . . . A A A A A A A A A A A A A    
1186	. . . . . . . . . . . . . . . A . . . . A A A A A A A A A A A A A    
1187	. . . . . . . . . . . . . . . G . . . . G G G G G G G G G G G G G    
1188	. . . . . . . . . . . . . . . C . . . . C C C C C C C C C C C C C    
1189	. . . . . . . . . . . . . . . T . . . . T T T T T T T T T T T T T    
1190	. . . . . . . . . . . . . . . A . . . . A A A A A A A A A A A A A    
1191	. . . . . . . . . . . . . . . T . . . . T T T T T T T T T T T T T    
1192	. . . . . . . . . . . . . . . A . . . . A A A A A A A A A A A A A    
1193	. . . . . . . . . . . . . . . T . . . . T T T T T T T T T T T T T    
1194	. . . . . . . . . . . . . . . T . . . . T T T T T T T T T T T T T    
1195	. . . . . . . . . . . . . . . T . . . . T T T T T T T T T T T T T    
1196	. . . . . . . . . . . . . . . T . . . . T T T T T T T T T T T T T    
1197	. . . . . . . . . . . . . . . G . . . . G G G G G G G G G G G G G    
1198	. . . . . . . . . . . . . . . T . . . . T T T T T T T T T T T T T    
1199	. . . . . . . . . . . . . . . . . . . . . A A A A A A A A A A A A    
1200	. . . . . . . . . . . . . . . . . . . . . T T T T T T T T T T T T    
1201	. . . . . . . . . . . . . . . . . . . . . G G G G G G G G G G G G    
1202	. . . . . . . . . . . . . . . . . . . . . T T T T T T T T T G G T    
1203	. . . . . . . . . . . . . . . . . . . . . . . A A . . A A A . . .    
1204	. . . . . . . . . . . . . . . . . . . . . . . T T . . T T T . . .    
1205	. . . . . . . . . . . . . . . . . . . . . . . A . . . A A A . . .    
1206	. . . . . . . . . . . . . . . . . . . . . . . G . . . G G G . . .    
1207	. . . . . . . . . . . . . . . . . . . . . . . C . . . T T T . . .
```
